# Nonhypoxic regulation and role of hypoxia-inducible factor 1 in aromatase inhibitor resistant breast cancer

**DOI:** 10.1186/bcr3609

**Published:** 2014-01-29

**Authors:** Armina A Kazi, Rabia A Gilani, Amanda J Schech, Saranya Chumsri, Gauri Sabnis, Preeti Shah, Olga Goloubeva, Shari Kronsberg, Angela H Brodie

**Affiliations:** 1 Department of Pharmacology and Experimental Therapeutics, University of Maryland, Baltimore, MD 21201, USA; 2 Department of Medicine, University of Maryland, Baltimore, MD 21201, USA; 3 School of Medicine, University of Maryland Marlene and Stewart Greenebaum Cancer Center, Baltimore, MD 21201, USA; 4 Division of Biostatistics and Bioinformatics Department of Epidemiology and Public Health, University of Maryland, Baltimore, MD 21201, USA; 5 Department of Biology, Loyola University Maryland, Baltimore, MD 21210, USA; 6 Department of Pharmacology and Experimental Therapeutics, University of Maryland School of Medicine, Health Science Facilities, Room 580G, 685 West Baltimore Street, Baltimore, MD 21201, USA

## Abstract

**Introduction:**

Although aromatase inhibitors (AIs; for example, letrozole) are highly effective in treating estrogen receptor positive (ER+) breast cancer, a significant percentage of patients either do not respond to AIs or become resistant to them. Previous studies suggest that acquired resistance to AIs involves a switch from dependence on ER signaling to dependence on growth factor-mediated pathways, such as human epidermal growth factor receptor-2 (HER2). However, the role of HER2, and the identity of other relevant factors that may be used as biomarkers or therapeutic targets remain unknown. This study investigated the potential role of transcription factor hypoxia inducible factor 1 (HIF-1) in acquired AI resistance, and its regulation by HER2.

**Methods:**

In vitro studies using AI (letrozole or exemestane)-resistant and AI-sensitive cells were conducted to investigate the regulation and role of HIF-1 in AI resistance. Western blot and RT-PCR analyses were conducted to compare protein and mRNA expression, respectively, of ERα, HER2, and HIF-1α (inducible HIF-1 subunit) in AI-resistant versus AI-sensitive cells. Similar expression analyses were also done, along with chromatin immunoprecipitation (ChIP), to identify previously known HIF-1 target genes, such as breast cancer resistance protein (BCRP), that may also play a role in AI resistance. Letrozole-resistant cells were treated with inhibitors to HER2, kinase pathways, and ERα to elucidate the regulation of HIF-1 and BCRP. Lastly, cells were treated with inhibitors or inducers of HIF-1α to determine its importance.

**Results:**

Basal HIF-1α protein and BCRP mRNA and protein are higher in AI-resistant and HER2-transfected cells than in AI-sensitive, HER2- parental cells under nonhypoxic conditions. HIF-1α expression in AI-resistant cells is likely regulated by HER2 activated-phosphatidylinositide-3-kinase/Akt-protein kinase B/mammalian target of rapamycin (PI3K/Akt/mTOR) pathway, as its expression was inhibited by HER2 inhibitors and kinase pathway inhibitors. Inhibition or upregulation of HIF-1α affects breast cancer cell expression of BCRP; AI responsiveness; and expression of cancer stem cell characteristics, partially through BCRP.

**Conclusions:**

One of the mechanisms of AI resistance may be through regulation of nonhypoxic HIF-1 target genes, such as *BCRP*, implicated in chemoresistance. Thus, HIF-1 should be explored further for its potential as a biomarker of and therapeutic target.

## Introduction

Breast cancer is the most prevalent form of cancer among women in the United States and second leading cause of cancer related deaths [1]. Approximately 70% to 80% of breast cancers express estrogen receptor (ER+) and, consequently, are estrogen-dependent in their growth. Endocrine/hormonal therapies have proven effective in treating ER + breast cancers. Selective estrogen receptor modulators (SERMS), such as tamoxifen, inhibit estrogen action on breast cancer cells by blocking ER + signaling. Alternatively, aromatase inhibitors (AIs; for example, letrozole, anastrozole, and exemestane) reduce circulating levels of estrogen by inhibiting the conversion of androgens to estrogen by the enzyme aromatase [2,3]. Comparing the efficacy of tamoxifen versus AIs, a number of clinical studies have shown that AIs are superior in terms of disease free survival, time to recurrence and prevention of contralateral breast cancer [4,5]. In the adjuvant setting, AIs are less toxic with minimal adverse effects compared to chemotherapy and provide protection against development of contralateral breast cancer. AIs are now first-line treatments for ER + breast cancer in post-menopausal women [6]. However, a significant percentage (range 30% to 65%) of patients either does not respond to AIs [7] or becomes resistant to them [8-10].

Studies from this lab and others suggest that resistance to AIs occurs after a switch from dependence on ER signaling to dependence on growth factor-mediated pathways, such as human epidermal growth factor receptor-2 (HER2), a member of the membrane epidermal growth factor receptor (EGFR) family of receptor tyrosine kinases, and insulin-like growth factor receptor (IGFR) [9-11]. Pre-clinical [12] and clinical [10] studies have explored HER2 inhibitors, trastuzumab and lapatinib, as treatments for letrozole-resistant breast cancer. Pre-clinically, our laboratory has shown that trastuzumab alone or in combination with letrozole decreased HER2 expression, restored ERα expression, and inhibited tumor growth of MCF-7Ca xenografts that became resistant to letrozole [13]. Clinically, it has been shown that lapatinib in combination with letrozole significantly increased progression-free survival in patients versus letrozole alone as first-line therapy for hormone receptor- and HER2-positive postmenopausal metastatic breast cancer [10,14]. However, studies with *de novo* HER2+ breast cancer (that is, not HER2+ breast cancer of acquired AI resistance) indicate that resistance can develop to HER2 inhibitors as well [15,16]. Thus, although it has yet to be studied, there may be a risk of developing resistance to second-line HER2 inhibitor therapy in patients who have already acquired resistance to first-line AI therapy. As a membrane receptor, HER2 can affect many cellular pathways, some of which may not be directly involved in the development of AI resistance. Targeting another factor downstream of HER2 that more directly mediates effects specific and essential to the development of AI resistance may be as effective as targeting HER2 itself, while not having the same level of risk of producing second-line acquired resistance. Currently, the mechanism by which HER2 is involved in AI resistance remains unclear. It is, therefore, important to: 1) further elucidate the HER2-mediated pathway that contributes to AI resistance, particularly characteristics associated with AI resistant breast cancer cells; and 2) identify other potential factors involved that may serve as novel molecular biomarkers and therapeutic targets.

One factor that may be involved in HER2-mediated AI resistance is HIF-1, a heterodimeric transcription factor made up of an inducible alpha (α) subunit and a constitutively expressed beta (β) subunit [17]. HIF-1α is normally kept low in cells by proteosomal degradation, but lack of sufficient oxygen levels (hypoxia, for example, 1% to 2% O_2_) prevents this degradation. This leads to increased intracellular HIF-1α protein levels, formation of HIF-1, and activation of HIF-1 target genes important for cell survival, metabolic adaptation and angiogenesis. Interestingly, HIF-1 expression and/or activation can also be regulated by growth factors, hormones and cytokines independent of O_2_ levels. For example, ERα- and HIF-1-mediated signaling pathways are known to interact antagonistically [18,19] and cooperatively [20-23]. EGFR and HER2, as well as kinase signaling pathways, such as the MAPK and PI3K/Akt pathways, have also been shown to regulate HIF-1α expression and activity [22,24,25].

The role of hypoxia-regulated HIF-1 in cancer has been well studied. This is particularly relevant to sizable tumors whose cancer cells are too distant from existing blood vessels to get enough oxygen and nutrients [26]. Hypoxia and/or HIF-1 have been implicated in increased patient mortality and disease progression [27]. Their involvement in tumor formation and metastasis [28,29], and regulation of cancer stem cells [28,30] and stem cell markers, such as breast cancer resistant protein (BCRP) [27,30,31], has also been demonstrated. However, nonhypoxic regulation of HIF-1 and its importance in cancer remains largely unknown. Specific to this study, the regulation and role of nonhypoxic HIF-1 in breast cancer cell resistance to AIs, specifically letrozole, has yet to be explored. Using a letrozole-resistant cell line developed from xenograft tumors in our laboratory, this current study tested the overall hypothesis that nonhypoxic HIF-1 is an essential factor in HER2-mediated letrozole resistance. More specifically, in letrozole-responsive tumors, the switch from ERα to HER2-dependent signaling increases HIF-1α expression, independent of nutrient or oxygen availability. HIF-1 then acts as a key transcription factor activating target genes involved in processes that promote letrozole resistance.

## Methods

### Cells and reagents

#### Cell culture

Cell lines (and their ER/HER2 status) used are listed in Table 1. MCF-7Ca cells obtained from the laboratory of Dr. Chen through institutional agreement (City of Hope, Duarte, CA, USA) are MCF-7 cells stably transfected with the human placental aromatase gene [32,33]. Aromatase is the enzyme that converts androgens to estrogen. Transfection of the aromatase gene does not affect ER/HER2 status, ER activation, or estrogen-dependence of MCF-7 cells, but it does allow MCF-7 cells to proliferate in response to androgens (for example, androstenedione) and be sensitive to growth inhibitor effects of aromatase inhibitors [33]. MCF-7Ca cells were maintained in (D)MEM 1× high glucose (Invitrogen, Grand Island, NY) supplemented with 5% fetal bovine serum (FBS), 1% penicillin/streptomycin (P-S), and 700 μg/mL geneticin selective antibiotic (G418). Long-term letrozole-treated (LTLTCa) cells, developed in our laboratory [34], are letrozole-resistant breast cancer cells isolated from MCF-7Ca mouse xenograft tumors treated for 56 weeks with letrozole and that have become resistant to the growth inhibitory effects of letrozole. LTLTCa cells were maintained in phenol red–free (PRF) modified Improved Minimal Essential Media (IMEM) (Invitrogen, Grand Island, NY) supplemented with 5% charcoal dextran-treated FBS (CDT-FBS), 1% P-S, 750 μg/mL G418, and 1 μM letrozole. LTLTCa cells can be compared to MCF-7Ca cells in experiments since they originated from the MCF-7Ca cell population and their expression of ERα and HER2 returns to levels similar to that of MCF-7Ca cells after letrozole withdrawal [35]. MCF-7 cells obtained from American Type Culture Collection (ATCC) were maintained in (D)MEM 1× high glucose (Invitrogen, Grand Island, NY) supplemented with 5% FBS and 1% P-S. Hc7 cells were developed in our laboratory; they are MCF-7 cells transfected with the *HER2* gene and overexpress HER2 [36]. Hc7 cells were maintained in (D)MEM 1× high glucose (Invitrogen, Grand Island, NY) supplemented with 5% FBS, 1% P-S, and 500 μg/ml hygromycin. AC1 cells are another set of MCF-7 cells stably transfected with the human placental aromatase gene [37]. Similar to MCF-7Ca cells, AC1 cells are ER+/HER2-, proliferate in response to estrogen or androstenedione, express the aromatase enzyme and are sensitive to aromatase inhibitors [38]. AC1 cells, however, were created in our laboratory rather than Dr. Chen’s, and they express higher levels of aromatase (data not shown). AC1 cells are maintained in (D)MEM 1x high glucose (Invitrogen, Grand Island, NY) supplemented with 5% FBS, 1% P-S, and 800 μg/mL G418. AC1-exemestane resistant (AC1-ExR) cells, developed in our laboratory, are exemestane-resistant cells isolated from AC1 mouse xenograft tumors treated for approximately 10 weeks with exemestane maintained in PRF modified IMEM (Invitrogen, Grand Island, NY) supplemented with 5% CDT-FBS, 1% P-S, 800 μg/mL G418, and 5 μM exemestane.

**Table 1:** ERα and HER2 status and aromatase inhibitor-sensitivity of cell lines used

**Cell line**	**ERα status**	**HER2 status**	**AI-sensitivity**
MCF-7Ca	**+**	**-**	Yes
LTLT-Ca	**-**	**+**	No (to letrozole)
MCF7	**+**	**-**	Yes
MCF7 / HER2	**+**	**+**	ND
AC1	**+**	**-**	Yes
AC1-Ex R	**+**	**+**	No (to exemestane)

ERα, estrogen receptor alpha; HER2, human epidermal growth factor receptor 2; ND: not determined.

For experiments determining the effect of oxygen tension on protein expression, MCF-7Ca and LTLTCa cells were plated in passage media and incubated either under normal (20% O_2_ at 37°C) or more physiological (5% O_2_; using a hypoxia chamber) cell culture conditions for 24 hours. MCF-7 cells used to generate MC-7Ca and AC1 cells were obtained from the ATCC and, thus, did not require ethical approval or patient consent.

#### Reagents

The following drugs were used: letrozole (Novartis, NY, USA); lapatinib (GlaxoSmithKline Pharmaceutical, Brentford, Middlesex, United Kingdom); trastuzumab (Genentech, San Francisco, CA); exemestane (Pfizer, NY, USA); cycloheximide (#C1988), actinomycin D (#A9415), and cobalt chloride (CoCl_2_; #C8661) (all from Sigma, St. Louis, MO). The following antibodies were used in western blot analyses: HER2 (#04-1127) and BCRP (EMD Millipore, Billerica, MA); HIF-1α (#610959; BD Biosciences); ERα (#8644; Cell Signaling Technology, Danvers, MA); phosphorylated and total ERK1/2, Akt (#4058 and #4685), mTOR (#2971 and #2972) and p70 S6 kinase (#9205 and #9202) all from Cell signaling Technology, Danvers, MA); and β-actin (#4970; Cell Signaling Technology, Danvers, MA).

### Western blot analysis

Plated cells were washed with ice-cold PBS and then lysed with radioimmunoprecipitation (RIPA) buffer containing protease (#11836145001) and phosphatase inhibitors (#4906837001) (both from Roche Applied Sciences, Indianapolis, IN) by sonication and incubation for 20 minutes at 4°C. Lysed samples were centrifuged at 14,000 rpm for 20 minutes at 4°C to collect protein lysates (supernatant). A total of 10 to 40 μg of protein underwent 10% SDS–polyacrylamide gel electrophoresis (SDS-PAGE) and was transferred to a polyvinylidene difluoride membrane (PVDF; #IPVH00010 Millipore, Billerica, MA). The resulting blots were probed with specific mouse or rabbit primary antibodies and either goat anti-mouse or –rabbit secondary antibodies conjugated to horseradish peroxidase (#17 2–1011 and#172-1019; Biorad, Hercules, CA), respectively. Blots were developed using SuperSignal West Pico Chemiluminescent Substrate (#34080; Thermo Scientific, Waltham, MA). Blots that were to be re-probed were stripped with Restore Western Blot Stripping Buffer (#21059; Thermo Scientific, Waltham, MA) for 40 minutes at room temperature prior to incubation with another primary antibody. Densitometry was performed on each blot using either ImageQuant or ImageJ.

### Reverse transcripase-polymerase chain rection

#### RNA extraction and reverse transcription (RT)

RNA was extracted and purified using the RNeasy Mini Kit (#74104; Qiagen, Valencia, CA). RNA was reverse transcribed to complementary DNA (cDNA) using 200U of Moloney murine leukemia virus reverse transcriptase (#28025013; Invitrogen, Grand Island, NY) and incubating at 37°C for one hour.

#### Real-time PCR

mRNA expression analyses were carried out by real-time PCR using a DNA Opticon system (MJ Research, Waltham, MA) and using DyNAmo SYBR green qPCR mix (New England Biolabs, Ipswich, MA). Standard curves were generated by serially diluting the sample expected to have the most amount of the PCR product. The yield of product for each unknown sample was calculated by applying its threshold cycle, or C(T), value to the standard curve using the Opticon Monitor analysis software (version 1.01, MJ Research, Waltham, MA). Values were normalized to corresponding 18S rRNA values and expressed as the fold increase relative to controls. Primers for HER2, HIF-1α, BCRP, GAPDH, BMI-1, Nanog and TWIST were obtained from (Sigma, St. Louis, MO or Qiagen Valencia, CA).

### Chromatin immunoprecipitation assay

For the *in vitro* chromatin immunoprecipitation (ChIP) assay, the treated cells were washed with DPBS and fixed with 1% formaldehyde/dulbecco's phosphate buffered saline (DPBS) for 10 minutes at 37°C, after which the cells were washed with ice-cold DPBS containing protease inhibitors. The cells were collected into 1 mL DPBS and pelleted by centrifugation at 6,000 rpm for five minutes at 4°C. The cell pellet was resuspended in nuclear lysis buffer (ChIP kit, #17–295 Millipore, Billerica, MA) and incubated on ice for 15 minutes. Samples were sonicated on ice for 7 × 10-second cycles, with 20-second pauses between each cycle. The sonicated samples were centrifuged at 14,000 rpm for 10 minutes at 4°C. The sonicated samples were diluted 1:10 with dilution buffer (ChIP kit) before being immunocleared in a solution containing protein A- or G-Sepharose slurry (#16-156 and #16-266, respectively; Millipore, Billerica, MA) in Tris/ethylenediaminetetraacetic acid (EDTA) buffer, salmon sperm DNA (#15632011; Invitrogen, Grand Island, NY), and normal mouse or rabbit serum (#M5905 and #R9133; Sigma, St. Louis, MO) for two hours at 4°C. Immunocleared supernatants were incubated overnight at 4°C with anti-HIF-1α antibody (#610959; BD Biosciences). Protein A- or G-Sepharose beads and salmon sperm DNA were then added and incubated for one hour at 4°C. The beads were then washed sequentially with 1 mL each of wash buffers (ChIP kit). The protein-DNA complexes were eluted by twice incubating the beads in an elution buffer for 10 minutes at room temperature with vigorous mixing. To separate immunoprecipitated protein and DNA, the pooled elutes were incubated at 65°C overnight. The DNA was purified using the QIAquick PCR Purification kit (#28106; Qiagen Valencia, CA). The yield of target region DNA in each sample after ChIP was analyzed by real-time PCR using primers for a region of BCRP promoter that contains a HIF-1 response element (Invitrogen, Grand Island, NY) or a negative control open reading frame (ORF)-free intergenic region (ChIP-qPCR Human IGX1A Negative Control primers (#GPH100001C(-)01A, Qiagen, Valencia, CA).

### Mammosphere assay

The mammosphere assay was performed using reagents from Stem Cell Technologies (Vancouver, CA), according to the manufacturer’s instructions. Single cells were suspended in complete Mammocult media according to the manufacturer’s instructions (#05620) and plated in ultra low attachment plates (#CLS3471; Corning, Tewksbury, MA) at a density of 10,000 to 20,000 cells/mL. Media were replenished every three days. Mammospheres were counted after at least seven days and up to three to four weeks. Spheres with a colony count of at least 50 cells were considered mammospheres.

### Statistical analysis

All experiments were performed two to three times, with multiple replicates at each time point (total of n = 4 to 6 independent samples). Thus, quantified values are means of n = 4 to 6 independent samples/group with standard deviations (SD). Statistical analyses were performed using Graph Pad Prism software and included: 1) a two-sided t-test to compare two groups (for example, MCF-7Ca versus LTLTCa); 2) a one-way analysis of variance (ANOVA) with Tukey’s adjustment to compare three or more groups (for example, different treatment types, time points, and so on); and 3) a two-way ANOVA with Bonferroni adjustment (for example, different cell types and treatments or genes). For mRNA stability, the linear mixed-effects models approach was used. To assure approximate normality, the logarithmic transformation was applied to the normalized value of mRNA, that is, for each cell line, mRNA expression at each actinomycin D time point was normalized using the corresponding vehicle-treated samples. Average mRNA expressions were estimated and compared at the pre-specified time-points (Additional file 1: Table S1.1 and Table S1.2), the trends over time were determined for HIF-α mRNA and BCRP mRNA. All required models’ diagnostics were performed. There were fixed effects for time, experiment, cell lines and interactions between time and cell lines. The models had hierarchical structure as repeated measurements were taken within a well, nested within time, cell line, and experiment. The compound symmetry was chosen as appropriate to model the variance structure of the random effects. Statistical tests were two-sided. Analyses were conducted using SAS (v.9.22, SAS Inc., NC, USA). The alpha level applied in all statistical analyses was *P* <0.05.

## Results

### LTLTCa cells have higher HIF-1α protein expression than MCF-7Ca cells under nonhypoxic conditions

**Figure 1:**
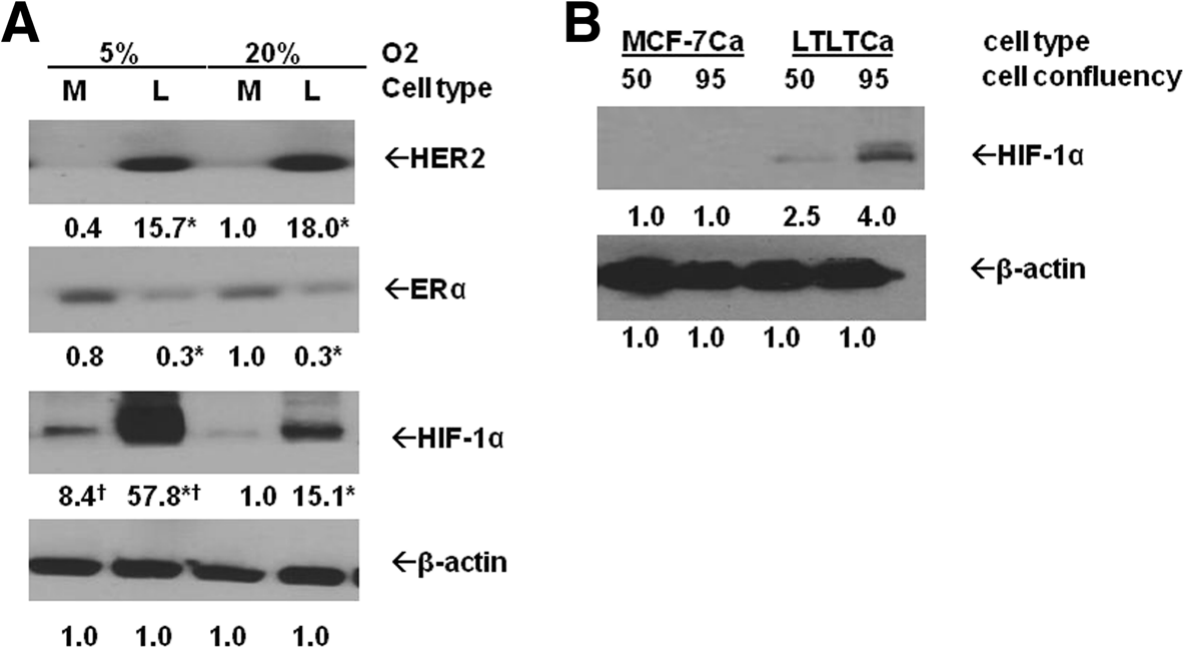
**Comparison of protein expression in parental MCF-7Ca and LTLTCa cells under different oxygen tension and cell confluency. A)** Parental MCF-7Ca and LTLTCa cells were plated and cultured in their respective passage media under either 5% O_2_ (*in vivo* normoxic/physiological conditions) or 20% O_2_ (normal, nonhypoxic cell culture conditions). Total protein was extracted and HER2, ERα, HIF-1α and β-actin were analyzed by Western blot analysis. Shown are representative blots and overall densitometry results of n = 6 independent cell samples/group. Densitometry results are expressed as mean fold-change in protein levels compared to MCF-7Ca cells in 20% O_2_ after normalization to β-actin (mean ± SD of n = 6 independent cell samples/group; *versus MCF-7Ca and † versus 20% O_2_; HER2 effect of cell type *P* = 0.0002, effect of % O_2_*P* = .5749, interaction between cell type and % O_2_*P* = .7337; ERα effect of cell type *P* <0.0001, effect of % O_2_*P* = .2879, interaction between cell type and% O_2_*P* = .2016; HIF-1α effect of cell type *P* = 0.0024, effect of % O_2_*P* = 0.0087, interaction between cell type and% O_2_*P* = 0.0413; two-way ANOVA). **B)** LTLTCa and parental MCF-7Ca cells were plated and cultured in their respective passage media at 1X or 2X density. Total protein was extracted when 2X density plates reached approximately 90% to 95% confluency, and, consequently, 1X density plates reached approximately 50% to 60% confluency. HIF-1α and β-actin protein were analyzed by Western blot. Densitometry results are expressed as mean fold-change compared to MCF-7Ca cells after normalization to β-actin. (mean ± SD, n = 6 independent cell samples/group; effect of cell confluency *P* = 0.0006, effect of cell type *P* < 0.0001, interaction between cell confluency and cell type *P* = 0.0006, two-way ANOVA). ANOVA, analysis of variance; ERα, estrogen receptor alpha; HER2, human epidermal growth factor receptor 2; HIF-1α, hypoxia inducible factor 1 α subunit; n, number; SD, standard deviation.

Previous studies have shown that a decrease in ERα and an increase in HER2 protein expression is associated with acquired AI-resistance [9-11], represented in this current study by LTLTCa cells. To determine whether HIF-1 expression is also associated with acquired AI-resistance, protein expression of the inducible HIF-1α subunit in LTLTCa and MCF-7Ca cells was determined. As expected, LTLTCa cells had 0.3 ± 0.02-fold ERα (*P* <0.0001); and 18.0 ± 5.5-fold HER2 (*P* = 0.002) protein levels compared to letrozole-sensitive parental MCF-7Ca cells under normal cell culture conditions (that is, nonhypoxic; 20% O_2_) (Figure 1A). LTLTCa cells also had 15.7 ± 5.9–fold (versus 1.0 ± 1.4 MCF-7Ca at 20% O_2_) higher basal levels of HIF-1α protein than their parental MCF-7Ca cells, which expressed little to no HIF-1α (Figure 1A).

Since oxygen levels in normal tissue [39,40], including the breast [26,41], range from 2% to 5%, and HIF-1α protein is known to be sensitive to O_2_ levels [17], protein expression at 5% O_2_ was also determined (Figure 1A). ERα and HER2 levels in both LTLTCa and MCF-7Ca cells remained unchanged when the percent O_2_ was reduced to more physiological levels. HIF-1α expression, in contrast and as expected, increased in both MCF-7Ca and LTLTCa cells (8.4 ± 3.1-fold and 57.8 ± 2.2-fold versus MCF-7Ca at 20% O_2_, Figure 1A). Nevertheless the fold differences in HIF-1α expression between LTLTCa cells and MCF-7Ca persisted and were significant.

Lastly, since HIF-1α protein expression can also be affected by cell density/confluency [42,43], protein expression in LTLTCa and MCF-7Ca cells at both approximately 50% and 95% confluencies were also analyzed. LTLTCa cells had higher levels of HIF-1α protein than MCF-7Ca cells under nonhypoxic conditions at both cell densities (*P* <0.0001, Figure 1B). Furthermore, while MCF-7Ca cells still had little or no HIF-1α protein at 95% confluency, LTLTCa cells exhibited a significant increase in HIF-1α (*P* = 0.0006; Figure 1B). These results suggest that: 1) letrozole-resistant LTLTCa cells basally and inherently have higher HIF-1α protein expression than letrozole-sensitive MCF-7Ca cells regardless of O_2_ levels or cell density; and 2) LTLTCa cells are more sensitive to inducers of HIF-1α expression, such as decreased O_2_ levels and cell density/confluency.

### HIF-1α expression in LTLTCa cells is due to increased protein synthesis

**Figure 2:**
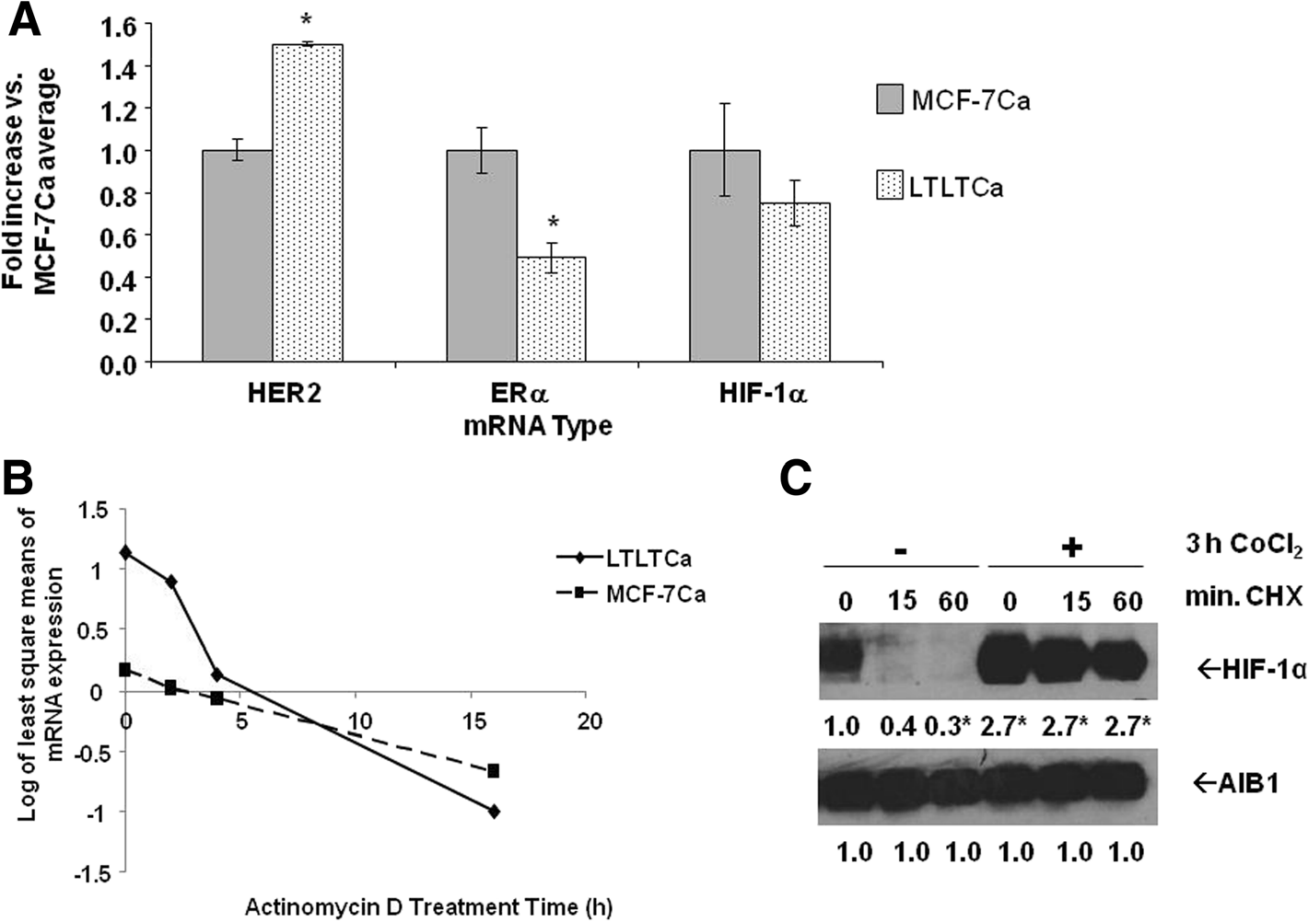
**Comparison of HIF-1α mRNA expression and stability in LTLTCa and MCF-7Ca cells. A)** LTLTCa and MCF-7Ca cells were plated and cultured in their respective passage media under normal cell culture (nonhypoxic) conditions. Total RNA was extracted and HER2, ERα, HIF-1α and 18S rRNA were analyzed by real-time RT-PCR analysis. Results shown are expressed as the mean fold-change in mRNA levels compared with MCF-7Ca cells after normalization to 18S rRNA (mean ± SD of n = 6 independent cell samples/group; *versus MCF-7Ca; effect of gene type *P* <0.0001, effect of cell type *P* = 0.1376, interaction between gene type and cell type *P* <0.0001; *MCF-7Ca versus LTLTCa for specific gene, *P* <0.0001; two-way ANOVA). **B)** LTLTCa and MCF-7Ca cells were treated with vehicle or 0.5 μg/ml actinomycin D for 0 to 16 hours. Total RNA was extracted and HIF-1α mRNA underwent real-time RT-PCR. Results are expressed as least square means of log transformed averages of mRNA expression at various timepoints (trend over time) after normalization to corresponding vehicle-treated samples and analysis by linear mixed effect model, adjusting for experiment, cell line and cell line*time interaction (means ± SD of n = 6 independent samples/group; *P* <0.001 for effect of cell line, time, their interaction and experiment). **C)** LTLTCa cells were treated with vehicle or 100 μM CoCl_2_ for three hours and then with 100 uM cycloheximide for 0 to 60 minutes. Whole cell protein was extracted and underwent Western blot for HIF-1α and AIB1 protein. Shown are representative blots and overall densitometry results of n = 6 independent cell samples/group. Densitometry results are expressed as mean fold-change in protein levels compared to vehicle-treated-0 minutes cycloheximide cells after normalization to AIB1 (mean ± SD of n = 6 independent cell samples/group; *versus no CoCl_2_-0 minutes CHX, *P* <0.0001, one-way ANOVA). ANOVA, analysis of variance; ERα, estrogen receptor alpha; HER2, human epidermal growth factor receptor 2; HIF-1α, hypoxia inducible factor 1 α subunit; n, number; SD, standard deviation.

Elevated HIF-1α protein expression in cells can result from increased protein stability and/or synthesis [44]. In LTLTCa cells, higher levels of HIF-1α may be due to increased protein synthesis (for example, increased mRNA translation to protein) for several reasons. First, unlike HER2 and ERα mRNA, HIF-1α mRNA expression was not significantly different between LTLTCa cells and MCF-7Ca cells (Figure 2A). This rules out increased *HIF-1α* gene transcription as the basis for increased HIF-1α protein. Second, overall through 16 hours of actinomycin D treatment HIF-1α mRNA was not more stable in LTLTCa cells compared to MCF-7Ca cells (Figure 2B and Additional file 1: Table S1.1). HIF-1α mRNA was more abundant in LTLTCa cells than MCF-7Ca cells prior to four hours of actinomycin D treatment, but it was less by sixteen hours (Figure 2B). Statistical analysis of HIF-1α mRNA expression over time in LTLTCa cells compared to MCF-7Ca cells showed significant effects of time, and cell line, and their interaction (*P* <0.001; linear mixed effect model of time regression analysis). Third, investigation of HIF-1α protein stability after treatment with the protein synthesis inhibitor cycloheximide with or without the HIF-1α protein stabilizer CoCl_2_[45], demonstrated that after addition of the protein synthesis inhibitor cycloheximide, HIF-1α protein in vehicle-treated LTLTCa cells rapidly degraded within 15 minutes (Figure 1C). In contrast, HIF-1α expression in CoCl_2_-treated LTLTCa cells was elevated by 2.8 ± 0.0-fold compared to 1.0 ± 0.3-fold in vehicle-treated cells (*P* <0.0001), and did not decrease through 60 minutes of cycloheximide treatment (2- to 2.8-fold at each time point, *P* <0.001) (Figure 1C). These protein results are consistent with what is known about the rapid proteosomal degradation of HIF-1α protein in nonhypoxic cells [44], and the effect of CoCl_2_ on HIF-1α protein stability [46]. These protein stability results further rule out increased protein stability as the basis for elevated HIF-1α levels in LTLTCa cells under nonhypoxic conditions.

### HER2-activated PI3K/Akt/mTOR pathway regulates HIF-1α expression in LTLTCa cells

**Figure 3:**
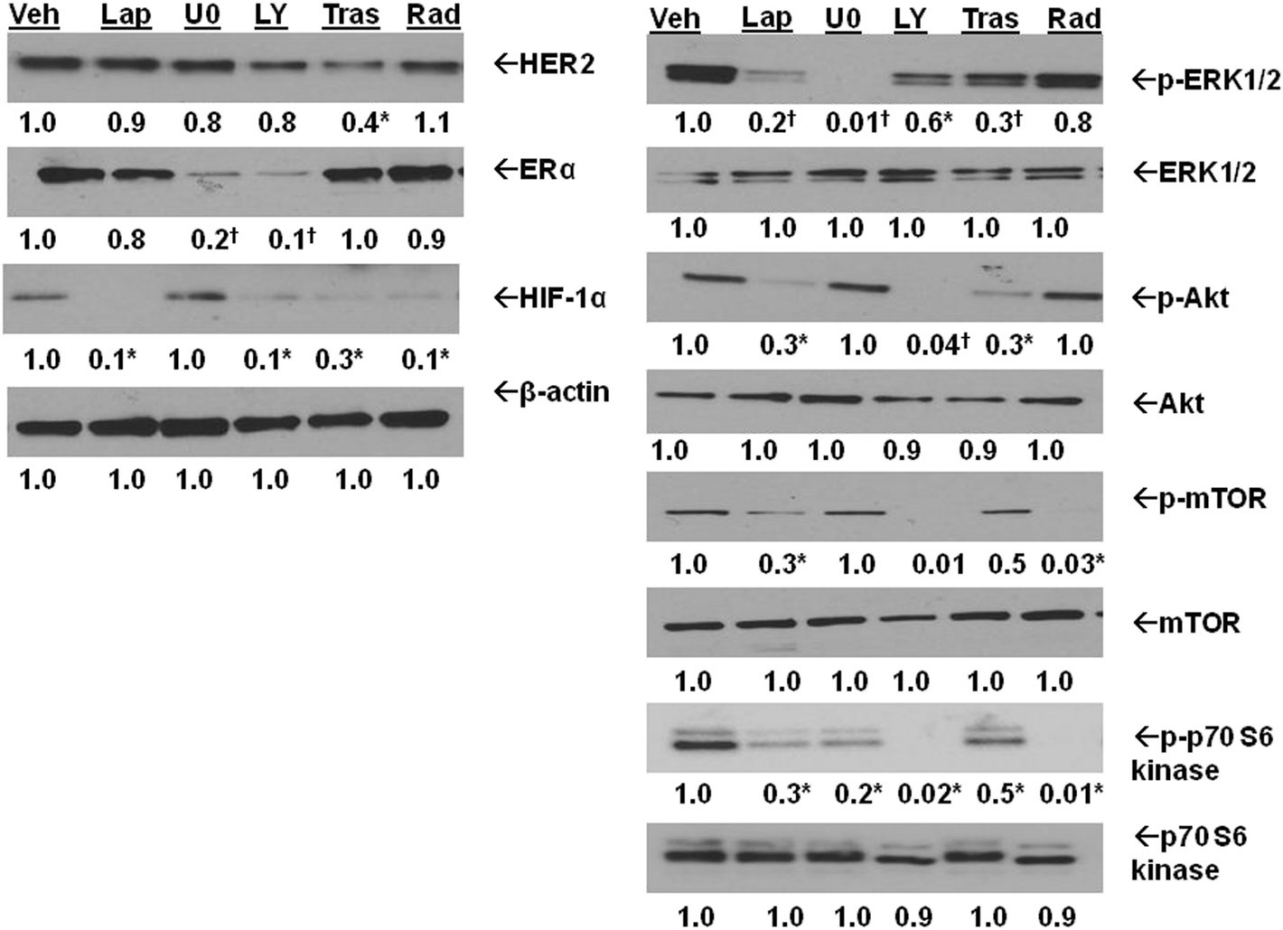
**Regulation of HIF-1α protein in LTLTCa cells. A)** LTLTCa cells were treated with either vehicle, 1 μM lapatinib, 20 μM MAPK pathway inhibitor U0126, 20 μM LY294002 PI3K pathway inhibitor, 500 μg/ml trastuzumab or 100 nM RAD001 for 24 hours. Total protein was extracted and HER2 (*P* = 0.128), phospho- and total-ERK1/2 (*P* <0.0001 for p-ERK), phospho- and total-Akt (*P* <0.0001 for p-Akt), phospho- and total mTOR (*P* = 0.0071), phospho- and total p70 S6 kinase (*P* <0.0001), ERα (*P* <0.0001), HIF-1α (*P* = 0.0003), and β-actin were analyzed by Western blot. Shown are representative blots and overall densitometry results of n = 6 independent cell samples/group. Densitometry results are expressed as mean fold-change in protein levels compared to vehicle-treated cells after normalization to β-actin (mean ± SD, n = 6 independent cell samples/group; *versus vehicle, *P* <0.05; † versus vehicle, *P* <0.001, one-way ANOVA). ANOVA, analysis of variance; ERα, estrogen receptor alpha; HER2, human epidermal growth factor receptor 2; HIF-1α, hypoxia inducible factor 1 α subunit; n, number; SD, standard deviation; mTOR, mammalian target of rapamycin.

Since LTLTCa cells have significantly higher HER2 protein and mRNA expression compared to MCF-7Ca cells ( [47], Figures 1A and 2A), this current study sought to determine whether endogenously overexpressed HER2 affects HIF-1α in LTLTCa cells. To do this, the effects of two types of HER2 inhibitors on HIF-1α were studied (Figure 3). Lapatinib is a HER2 kinase inhibitor that does not affect HER2 expression but does decrease HER2 activation of downstream kinase pathways (for example, MAPK, PI3K/Akt/mTOR pathway). Trastuzumab is a HER2 monoclonal antibody that decreases HER2 expression and its activation of downstream kinase pathways. As expected, only trastuzumab significantly reduced HER2 protein expression (0.4 ± 0.05 versus 1 ± 0.2 vehicle-treated), but both lapatinib and trastuzumab inhibited activation of the MAPK (0.2 ± 0.1-fold and 0.3 ± 0.01-fold, respectively, versus 1 ± 0.2-fold vehicle-treated of p-ERK1/2, *P* <0.05) and PI3K/Akt pathways (0.2 ± 0.2-fold and 0.3 ± 0.08-fold versus 1 ± 0.09-fold vehicle of p-Akt, *P* <0.01) (Figure 3). Both inhibitors also significantly decreased HIF-1α protein expression in LTLTCa cells (0.2 ± 0.2-fold and 0.4 ± 0.2-fold, respectively, versus of 1 ± 0.1-fold vehicle-treated, *P* <0.001) (Figure 3).

Since both the MAPK and PI3K/Akt/mTOR pathways are activated by HER2 and known to regulate HIF-1α expression and activity [44,48,49], the effect of specific inhibition of each pathway on HIF-1α expression was also studied (Figure 3). As expected, the MAPK inhibitor U0126 effectively decreased p-ERK1/2 protein expression (0.01 ± 0.01-fold versus 1 ± 0.2-fold vehicle-treated, *P* <0.001), and the PI3K inhibitor LY294002 decreased p-Akt (0.01 ± 0.01-fold versus 1 ± 0.09-fold vehicle-treated), downstream Akt target p-mTOR (0.5 ± 0.2-fold versus 1 ± 0.2-fold vehicle-treated), downstream mTOR target p-70 S6 kinase (0.02 ± 0.02-fold versus 1 ± 0.1-fold vehicle-treated). Also as expected, the mTOR inhibitor Rad001 decreased phosphorylation of mTOR (0 ± 0.01 versus 1 ± 0.1-fold vehicle) and p70 S6 kinase (0.02 ± 0.02-fold versus 0.1 ± 0.1-fold vehicle) without affecting upstream Akt (Figure 3). HIF-1α protein expression was significantly decreased in LTLTCa cells with LY294002 (0.1 ± 0.1-fold versus 1 ± 0.1-fold vehicle-treated) and Rad001 (0.1 ± 0.06-fold versus 1 ± 0.1-fold vehicle-treated), but not by U0126. Overall, these results indicate that HER2 activation of the PI3K/Akt/mTOR pathway induces HIF-1α expression. They also suggest that the HER2-activated MAPK pathway in LTLTCa cells has distinct functions from that of the PI3K/Akt/mTOR pathway.

### HIF-1α involvement in HER2 regulation of BCRP expression

**Figure 4:**
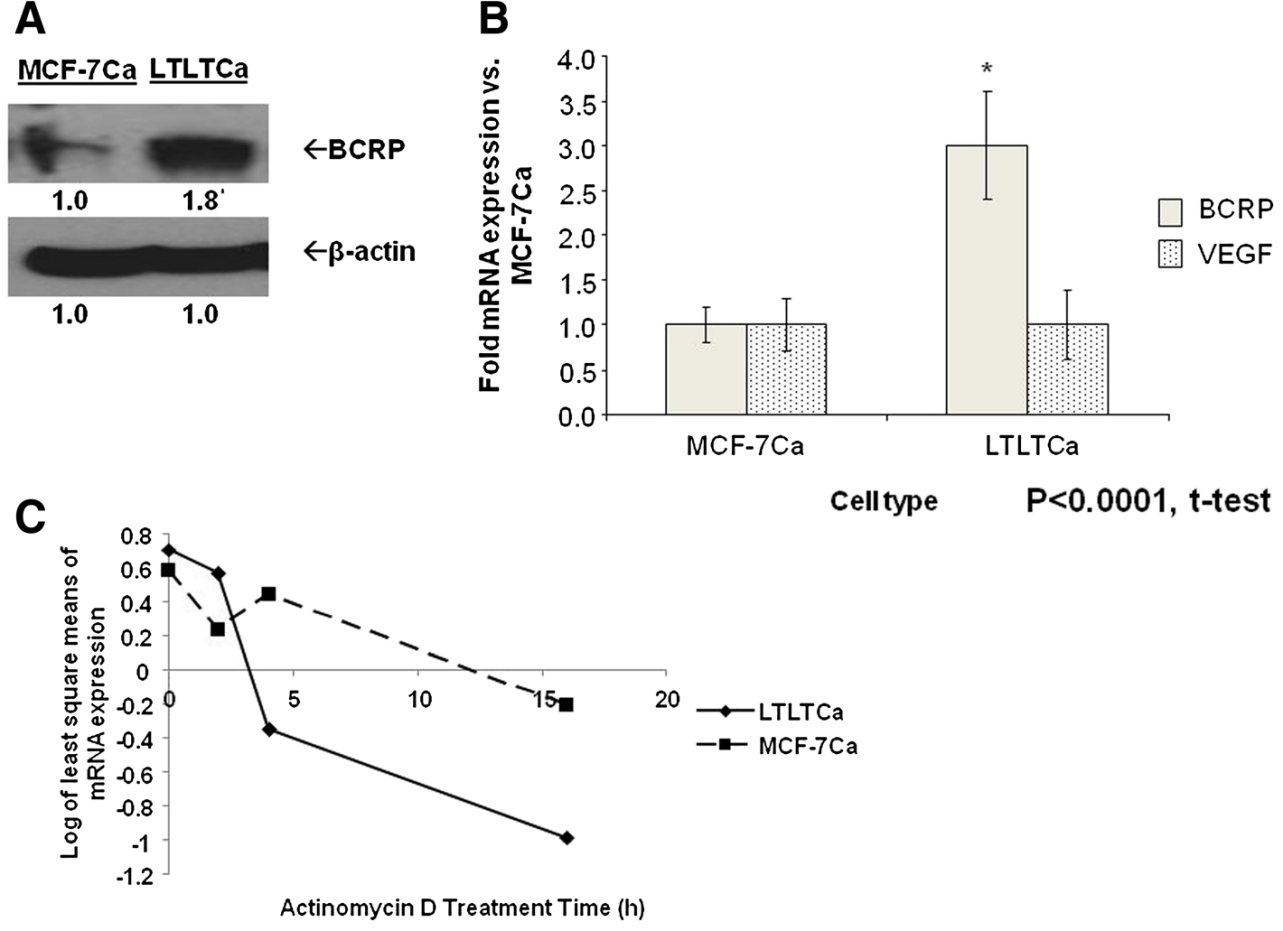
**Comparison of BCRP protein and mRNA expression and stability in LTLTCa and MCF-7Ca cells.** LTLTCa and parental MCF-7Ca cells were plated and cultured in their respective passage media under normal cell culture (nonhypoxic) conditions. **A)** Total protein was extracted and BCRP and β-actin were analyzed by Western blot analysis. Densitometry results are expressed as fold-change in protein levels compared to MCF-7Ca cells after normalization to β-actin (mean ± SD, n = 6 independent cell samples/group; *versus MCF-7Ca, *P* <0.0001, two-sided t test). **B)** Total RNA was extracted and BCRP mRNA, VEGF mRNA and 18S rRNA were analyzed by real-time RT-PCR analysis. Results are expressed as the fold-change in mRNA levels compared with MCF-7Ca cells after normalization to 18S rRNA (mean ± SD, n = 6 independent cell samples/group; *versus MCF-7Ca, *P* <0.0001, two-tailed t-test). **C)** LTLTCa and MCF-7Ca cells were treated with vehicle or 0.5 μg/ml actinomycin D for 0 to 16 hours. Total RNA was extracted and BCRP mRNA was analyzed by real-time RT-PCR. Results are expressed as least square means of log transformed averages of mRNA expression at various timepoints (trend over time) after normalization to corresponding vehicle-treated samples, and analysis by linear mixed effect model adjusting for experiment, cell line, and cell line*time interaction (mean ± SD of n = 6 independent cell samples/group; *versus MCF-7Ca; effect of gene type *P* = 0.0025, effect of cell type *P* = .3749, interaction between gene type and cell type *P* = 0.0025; two-way ANOVA). ANOVA, analysis of variance; BCRP, breast cancer resistant protein; n, number; SD, standard deviation; VEGF, vascular endothelial growth factor.

As a transcription factor, HIF-1 may be mediating the effects of HER2 on target genes that contribute to the LTLTCa cell phenotype. One such gene may be the breast cancer resistance protein (*BCRP*), an efflux transporter protein and stem cell marker implicated in cancer cell chemoresistance [50,51]. *BCRP* is also known to be a HIF-1 target gene [31]. Recently, findings from our laboratory have shown that BCRP protein is overexpressed in LTLTCa cells compared to MCF-7Ca cells and that BCRP is important in stem cell characteristics of LTLTCa (for example, mammosphere formation, side population percentage) [52]. This current study confirms the overexpression of BCRP protein in LTLTCa cells, and further demonstrates that BCRP mRNA expression (3 ± 0.6-fold versus 1 ± 0.2-fold vehicle-treated, *P* <0.0001, one-way ANOVA) is also elevated compared to MCF-7Ca cells (Figure 4A-B). BCRP mRNA stability was also compared between the two cell types. Consistent with findings of others in MCF-7 cells [53], BCRP mRNA is fairly stable through 16 hours of actinomycin D treatment in MCF-7Ca cells (Figure 2C). Overall, BCRP mRNA in LTLTCa cells was not significantly more stable than in MCF-7Ca (Figure 4C). Statistical analysis of BCRP mRNA expression over time in LTLTCa cells compared to MCF-7Ca cells showed significant effects of time, but not cell line (effect of time *P* < 0.001; effect of cell line at *P* = 0.049; linear mixed effect model of time regression analysis). Thus, overexpression of BCRP in LTLTCa cells is attributed to increased synthesis at the gene transcription level.

**Figure 5:**
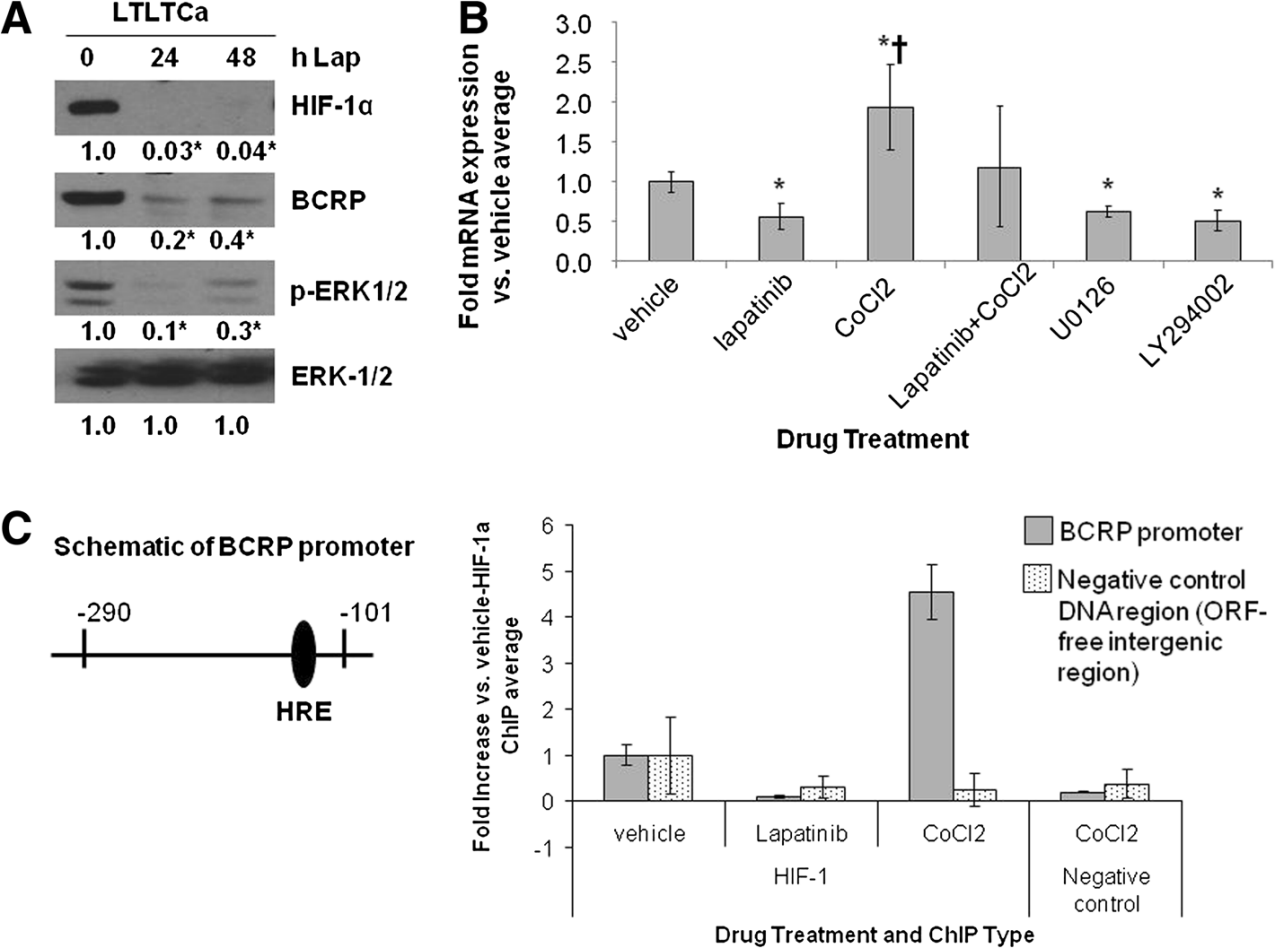
**Effect of lapatinib and CoCl**_2 _**on BCRP protein and mRNA expression and on HIF-1α binding to the BCRP promoter. A-B), A)** LTLTCa cells were treated with 1 μM lapatinib for 0 to 48 hours. Total protein was extracted and underwent Western blot for HIF-1α, BCRP, p-ERK1/2, and ERK. Shown are representative blots and overall densitometry results of n = 6 independent cell samples/group. Densitometry results are expressed as mean fold-change compared to 0 hours after normalization to ERK (mean ± SD of n = 6 independent cell samples/group; *versus 0 hours lapatinib; *P* = 0.0004 for ERK for HIF-1α, *P* = 0.0017 for BCRP, *P* = 0.0009 for phospho-ERK1/2, *P* = 1 for ERK-1/2; one-way ANOVA). **B)** Total RNA was extracted and underwent real-time RT-PCR for BCRP mRNA and 18S rRNA. Real-time results are the mean fold-change in mRNA levels compared with vehicle after normalization to 18S rRNA (mean ± SD of n = 6 independent cell samples/group; *versus vehicle, *P* <*0*.05; † versus lapatinib, U0126 or LY294002, *P* <0.01; overall *P* <*0*.0001, one-way ANOVA). **C)** LTLTCa were treated with vehicle, 1 μM lapatinib, and/or 100 μM CoCl_2_ for 24 hours. Protein-DNA complexes from LTLTCa cells were analyzed by ChIP analysis. Immunoprecipitation was done either with HIF-1α antibody or an equivalent volume of normal mouse serum (negative control). Primers for either the -290 to -101 region of the human BCRP promoter, which contains the HRE to which HIF-1 binds, or ORF-free intergenic region (negative control DNA region) were used for real-time PCR. Results are the mean fold increase compared with vehicle-treated cells after normalization to input samples of each (means ± SD, n = 6 independent cells sample/group; *versus vehicle-HIF-1 IP; *P* = 0.004 for BCRP promoter, *P* = .2972 for negative control ORF-free intergenic region; one-way ANOVA). ANOVA, analysis of variance; BCRP, breast cancer resistant protein; ChIP, chromatin immunoprecipitation; HIF-1α, hypoxia inducible factor 1 α subunit; n, number; SD, standard deviation.

In order to elucidate the factors and pathways involved in regulating BCRP expression, the effects of the HER2 kinase inhibitor lapatinib, HIF-1α stabilizer CoCl_2_, and/or specific kinase pathway inhibitors U0126 (MAPK pathway) and LY294002 (PI3K/Akt pathway) on BCRP protein or mRNA expression were assessed (Figure 5A-B). Lapatinib reduced both BCRP protein (0.2-fold versus 1.0-fold vehicle, *P* <0.01) and mRNA (0.6 ± 0.2-fold versus 1.0 ± .0.02-fold vehicle-treated, *P* <0.01, one-way ANOVA) levels in LTLTCa cells. This decrease correlated with lapatinib’s inhibitory effects on HIF-1α and p-ERK1/2 expression. Inhibition of either the MAPK or PI3K/Akt pathways also resulted in decreased BCRP mRNA levels (0.7 ± 0.05-fold and 0.55 ± 0.08-fold versus 1.0 ± .0.02 vehicle-treated, *P* <0.01, respectively; Figure 5B). CoCl_2_ treatment conversely increased BCRP mRNA in LTLTCa cells. Co-treatment with lapatinib and CoCl_2_ resulted in BCRP mRNA levels that tended to be intermediate of lapatinib-inhibited and CoCl_2_-induced levels, but not significantly different from either one and from vehicle (Figure 5B).

Although results in Figure 5A-B suggested that HER2, via the MAPK and PI3K/Akt pathways, and HIF-1α are both involved in regulating BCRP expression in LTLTCa cells, these expression analyses did not test whether HIF-1α actually mediates the effects of HER2 on target genes. ChIP analysis was, therefore, performed to determine HIF-1α binding to the BCRP promoter under basal, nonhypoxic conditions and after lapatinib or CoCl_2_ treatment. Real-time PCR analysis of immunoprecipitated DNA after ChIP showed that under basal, nonhypoxic conditions HIF-1α was bound to a hypoxia-response element (HRE)-containing region of the BCRP promoter in LTLTCa cells (Figure 5C). CoCl_2_ significantly increased HIF-1α binding to the BCRP promoter, but lapatinib treatment prevented this binding (versus 1 ± 1.2-fold vehicle-treated, *P* = 0.004, one-way ANOVA). Specificity of immunoprecipitation was confirmed by the lack of immunoprecipitated DNA in the negative IP control samples, as well as on the negative control DNA (*P* = 0.2972, one-way ANOVA). In addition, samples in the BCRP promoter PCR (excluding input) amplified at cycles 20 to 30, while samples in the negative control DNA region PCR (excluding input) amplified at cycles 32 to 40. Correlation between HIF-1α binding to the BCRP promoter and changes in BCRP mRNA and protein expression in the absence or presence of lapatinib suggests that HER2-regulated HIF-1 is involved in *BCRP* gene expression in LTLTCa cells.

Such regulation, however, does not appear to be relevant to all known HIF-1 target genes. Vascular endothelial growth factor (*VEGF*), another known HIF-1 target gene and important therapeutic target in cancer [17], is not upregulated in LTLTCa cells compared to MCF-7Ca cells (Figure 2B). Also, despite being induced by CoCl_2_, VEGF mRNA expression was not sensitive to lapatinib (data not shown).

### Effect of specific inhibition of HIF-1α on BCRP

**Figure 6:**
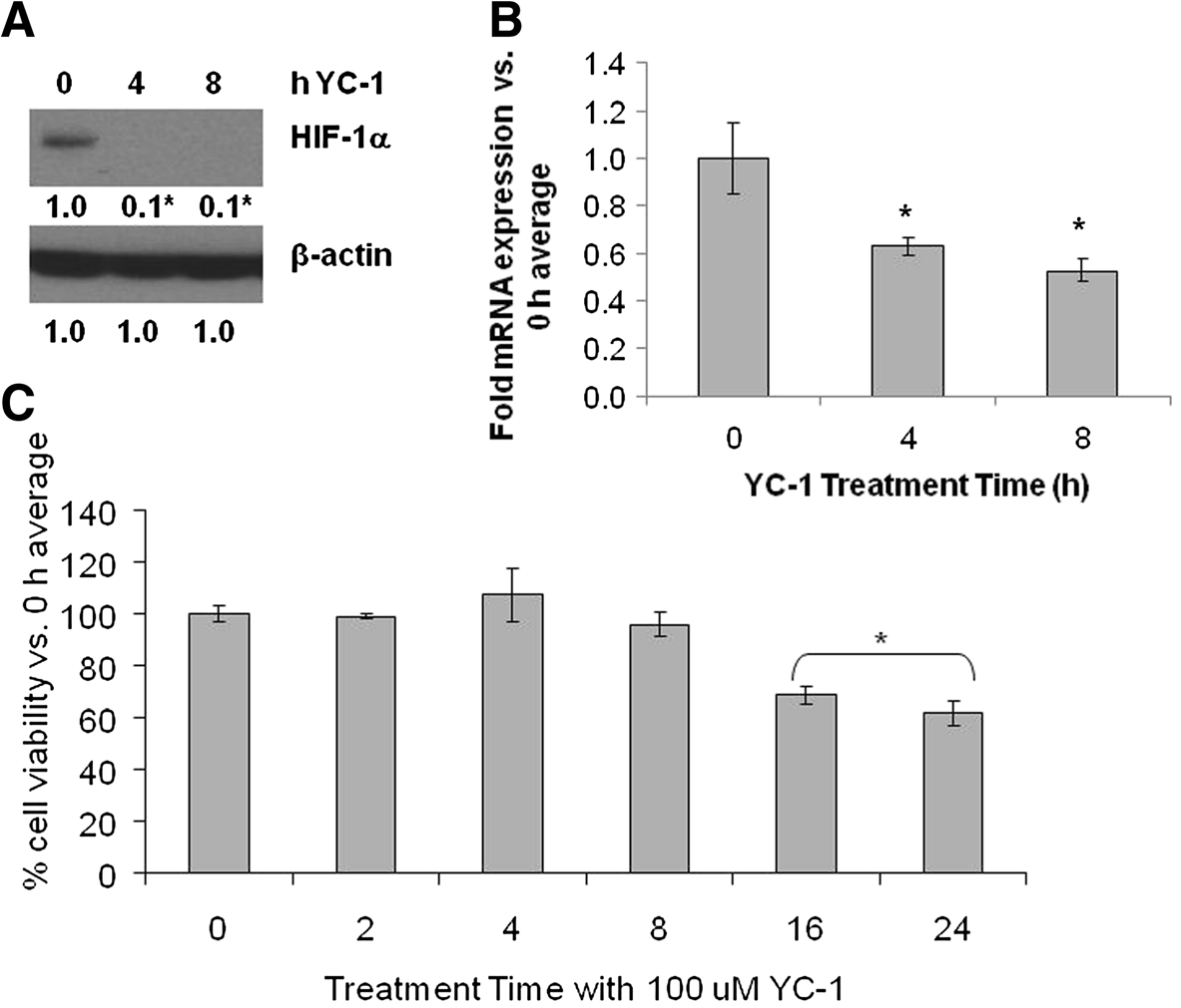
**Effect of YC-1 on HIF-1α and BCRP expression and cell viability in LTLTCa cells.** LTLTCa cells were treated with 100 μM YC-1 for 0 to 24 hours and effects on HIF-1α **(A)** and BCRP **(B)** expression and cell viability **(C)** were determined. **A)** After 0 to 4 hours of YC-1 treatment, total protein was extracted and HIF-1α and β-actin were analyzed by Western blot analysis. Shown are representative blots and overall densitometry results of n = 6 independent cell samples/group. Densitometry results are expressed as mean fold-change in protein levels compared to 0 hours after normalization to β-actin (mean ± SD of n = 6 independent cell samples/group; *versus 0 hours YC-1, *P* <0.001; overall *P* <0.0001, one-way ANOVA). **B)** After 0 to 8 hours YC-1 treatment, total RNA was extracted and BCRP mRNA and 18S rRNA were analyzed by real-time RT-PCR analysis. Real-time results are expressed as the mean fold-change in mRNA levels compared with vehicle after normalization to 18S rRNA (mean ± SD, n = 6 independent cell samples/group; *versus 0 hours YC-1, *P* <0.001; overall *P* <0.0001, one-way ANOVA). **C)** Viability of cells was measured by MTT assay after 0 to 24 hours treatment with YC-1. Results are expressed as mean percent of 0 hours average (mean ± SD, n = 4 independent cell samples/group; *versus 0 hours, *P* <0.001; overall *P* <*0*.0001, one-way ANOVA). ANOVA, analysis of variance; BCRP, breast cancer resistant protein; HIF-1α, hypoxia inducible factor 1 α subunit; MTT, 3-[4,5-dimethylthiazol-2-yl]-2,5 diphenyl tetrazolium bromide; n, number; SD, standard deviation.

**Figure 7:**
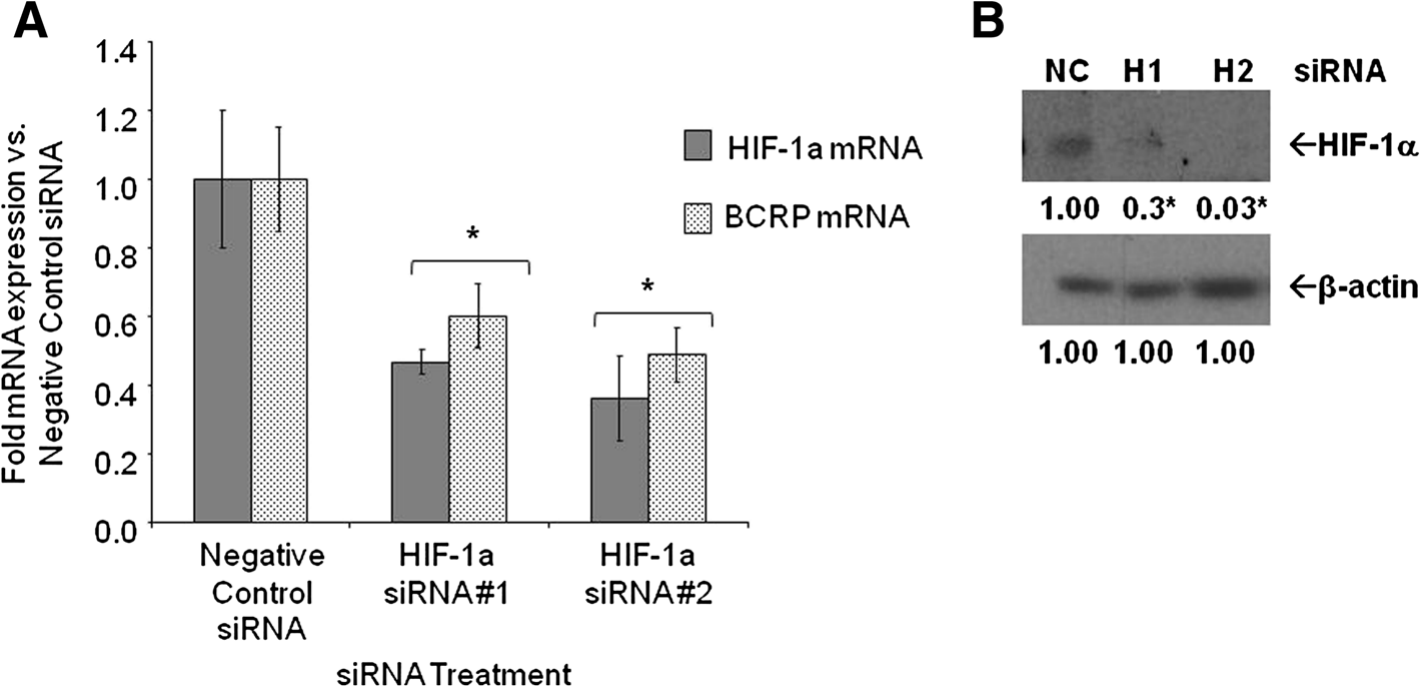
**Effect of HIF-1α siRNA on mRNA expression in LTLTCa cells. A)** LTLTCa cells were plated in passage media and then treated with two siRNAs for HIF-1α for 48 hours. Total mRNA was extracted and HIF-1α and BCRP mRNA, and 18S rRNA were analyzed by real-time RT-PCR. Real-time results are expressed as the mean fold-change in mRNA levels compared with negative control after normalization to 18S rRNA (mean ± SD, n = 6 independent samples/group; *versus negative control, *P* = 0.0057 for HIF-1α; *P* = 0.0026 for BCRP; one-way ANOVA). **B)** LTLTCa cells were plated in passage media and then treated with two siRNAs for HIF-1α for 48 hours. Total protein was extracted and HIF-1α and β-actin protein were analyzed by Western blot. Shown are representative blots and overall densitometry results of n = 6 independent cell samples/group. Densitometry results are expressed as mean fold-change in protein levels compared to negative control after normalization to β-actin (mean ± SD, n = 6 independent cell samples/group; *versus negative control (NC), *P* <0.0001, one-way ANOVA). ANOVA, analysis of variance; BCRP, breast cancer resistant protein; HIF-1α, hypoxia inducible factor 1 α subunit; n, number; SD, standard deviation.

To further support a connection between HIF-1α and BCRP, HIF-1α expression in LTLTCa cells was specifically inhibited by either YC-1, a known pharmacological inhibitor of HIF-1α [54,55] or siRNA. Similar to observations with lapatinib treatment, HIF-1α protein and BCRP mRNA expression were significantly decreased (0.1 ± 0.1-fold versus 0.1 ± 0.3-fold vehicle-treated and 0.5 ± 0.05-fold versus 1 ± 0.15-fold vehicle treated *P* <0.0001, respectively) in LTLTCa cells within eight hours of YC-1 treatment (Figure 6A-B). This correlated with a 30% to 40% decrease in LTLTCa cell viability by 16 and 24 hours, respectively (Figure 6C). Specific inhibition of HIF-1α expression by siRNAs also significantly decreased both HIF-1α mRNA (approximately 0.4 ± 0.04-fold versus 1 ± 0.2-fold negative control siRNA, *P* = 0.0057, one-way ANOVA) and protein (0.3 ± 0.1-fold to 0.03 ± 0.1-fold versus 1 ± 0.05-fold negative control, *P* <0.0001, one-way ANOVA), as well as BCRP mRNA (0.4- to 0.6-fold versus 1 ± 0.2-fold negative control siRNA, *P* <0.0001, one-way ANOVA) expression after 48 hours (Figure 7A-B).

### Correlation between HER2, HIF-1α, and BCRP in HER2-transfected cells and another AI-resistant cell line

**Figure 8:**
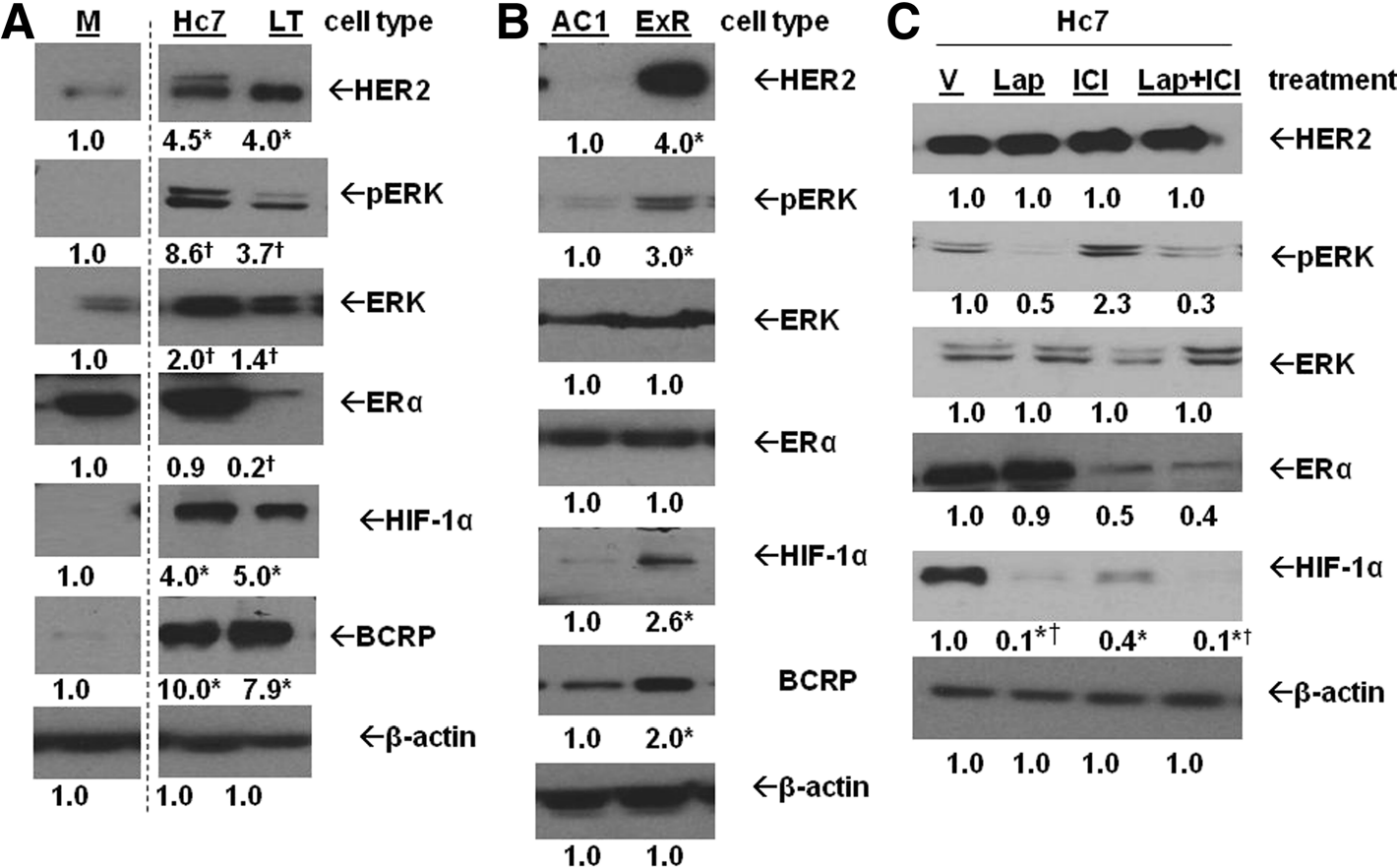
**Protein expression in HER2+ cells and exemestane-resistant cells. A)** MCF-7Ca (M), Hc7 and LTLTCa (LT) cells were plated in their respective passage media. Total protein was extracted and HER2, phosphorylated- and total-ERK, ERα, HIF-1α, BCRP and β-actin protein were analyzed by Western blot. Shown are representative blots and overall densitometry results of n = 6 independent cell samples/group. Densitometry results are expressed as mean fold-change compared to MCF-7Ca after normalization to ERK (mean ± SD of n = 6 independent cell samples/group; *versus MCF-7Ca, *P* <0.05; † versus MCF-7Ca, *P* <0.001, one-way ANOVA). Dashed lines indicate omitted lane in between M and Hc7 of the same blots. **B)** AC1 (AC1) and AC1-ExR (ExR) cells were plated in their respective passage media. Total protein was extracted and HER2, phosphorylated- and total-ERK, ERα, HIF-1α and β-actin protein were analyzed by Western blot. Shown are representative blots and overall densitometry results of n = 6 independent cell samples/group. Densitometry results are expressed as mean fold-change compared to vehicle-treated cells after normalization to β-actin (mean ± SD of n = 6 independent cell samples/group; *versus vehicle, P <0.0001, two-sided t-test). **C)** MCF-7/HER2 cells were treated with either vehicle (V), 1 μM lapatinib (Lap), 100 nM ICI 182,780 (ICI) or 1 μM lapatinib + 100 nM ICI 182,780 (Lap + ICI) for 24 hours. Total protein was extracted and HER2, phospho- and total-ERK1/2, ERα, HIF-1α and β-actin were analyzed by Western blot. Shown are representative blots and overall densitometry results of n = 6 independent cell samples/group. Densitometry results are expressed as mean fold-change compared to vehicle-treated cells after normalization to β-actin (mean ± SD of n = 6 independent cell samples/group; *versus vehicle, *P* <0.0001, one-way ANOVA). ANOVA, analysis of variance; BCRP, breast cancer resistant protein; ERα, estrogen receptor alpha; HER2, human epidermal growth factor receptor 2; HIF-1α, hypoxia inducible factor 1 α subunit; n, number; SD, standard deviation.

To further confirm the role of HER2 in regulating HIF-1α and BCRP and to determine if ERα is also involved, protein expression in Hc7 cells, ERα + MCF-7 cells transfected with *HER2* gene was also studied. Similar to ERα-/HER2+ LTLTCa cells, Hc7 cells overexpressed phospho-ERK, HIF-1α and BCRP protein expression compared to ERα+/HER2-parental MCF-7 cells (Figure 8A). Furthermore, HER2 inhibition by lapatinib decreased HIF-1α protein levels in Hc7 cells (0.1 ± 0.1-fold versus vehicle, *P* <0.0001; Figure 8B). Interestingly, inhibition of ERα alone by the ERα antagonist ICI 182,780 also reduced HIF-1α levels, but its effect on the protein level was significantly less than that of lapatinib alone or lapatinib and ICI182,780 in combination (Figure 8C). Another AI-resistant cell line, exemestane-resistant AC1-ExR breast cancer cells, was also analyzed. Despite retaining ERα, AC1-ExR cells also showed higher HER2, HIF-1α and BCRP protein levels. Overall, these results further indicate that increased HER2 and HER2-activated kinase pathways correlate with increased HIF-1α. They also indicate that while ERα can play a role in regulating HIF-1, as has been suggested by other studies [22], HER2 is likely to be the more important factor in the cells studied.

### Functional importance of HIF-1α in LTLTCa cells

#### Effect of HIF-1α inhibition on LTLTCa cells

Lastly, the functional importance of HIF-1 to the letrozole-resistant cell phenotype was explored. In cancer cells, hypoxia and HIF-1 are known to be involved in increased cell survival, chemoresistance [56,57], resistance to apoptosis [58] and maintenance of cancer stem cell characteristics [59,60]. Previous findings from our laboratory [52] have already demonstrated that letrozole resistance and cancer stem cell characteristics of LTLTCa cells are reduced by inhibition of HER2 and/or BCRP. Although Gilani *et al*. did not specifically test for the involvement of HIF-1, results of this study combined with those of our current study demonstrating the HER2-HIF-1-BCRP pathway, supports a role for HIF-1 in determining the letrozole-resistant cell phenotype.

**Figure 9:**
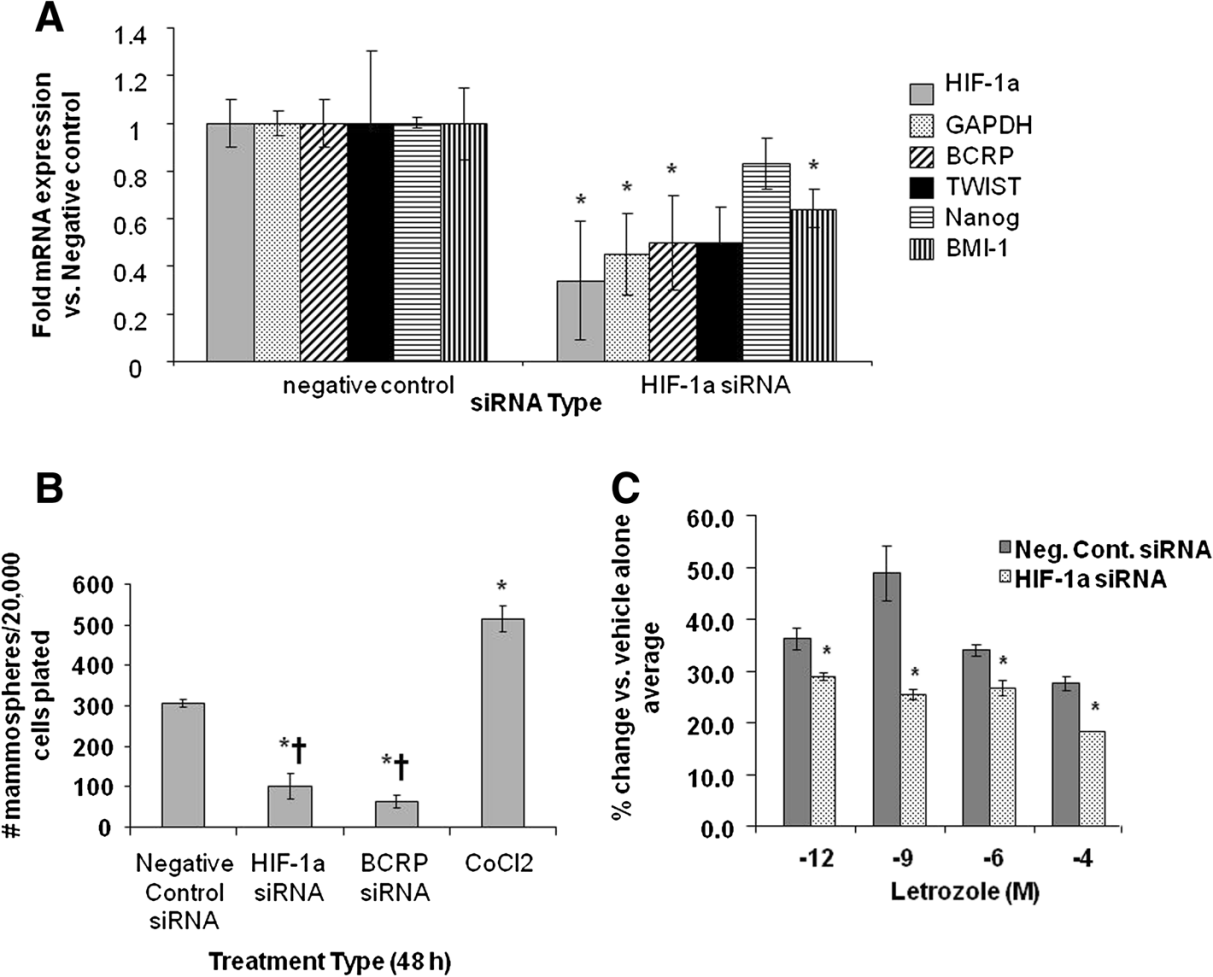
**Effect of HIF-1α and/or BCRP siRNA on mammosphere formation and cell proliferation in LTLTCa cells. A)** LTLTCa cells were treated with either negative control siRNA or HIF-1α siRNA for 48 hours. Total mRNA was extracted and HIF-1α, BCRP, GAPDH, Nanog, BMI-1 and TWIST mRNA, and 18S rRNA were analyzed by real-time RT-PCR. Real-time results are expressed as the fold-change in mRNA levels compared with negative control after normalization to 18S rRNA (mean ± SD, n = 6 independent cell samples/group; *versus vehicle; *P* = 0.0.132 HIF-1; *P* = 0.0058 GAPDH, *P* = 0.0377 BCRP, *P* = 0.0612 TWIST, *P* = 0.058 Nanog, *P* = 0.0214 BMI-1; two-sided t-test). **B)** LTLTCa cells were plated in passage media and then treated with negative control siRNA, HIF-1α siRNA, BCRP siRNA, or 100 μM CoCl_2_ for 48 hours. Cells were then collected and resuspended in mammosphere media on low-attachment cell culture wells. Results are expressed as number of mammospheres counted per 20,000 cells plated (mean ± SD, n = 6 independent cell samples/group; *versus negative control, *P* <0.001; † versus CoCl_2_, *P* < 0.001; overall *P* <0.0001, one-way ANOVA). BCRP siRNA confirmed to decrease BCRP expression (0.35- and 0.15-fold versus negative control, *P* <0.01, one-way ANOVA; data not shown). **C)** Viability of the cells was measured by the MTT assay after 48 hours treatment with negative control or HIF-1 alpha siRNA and subsequently 6 day treatment with increasing doses of letrozole. Results are expressed as percent of 0 μM letrozole (vehicle) (mean ± SD, n = 4 independent cell samples/group; *versus 0 μM letrozole, *P* <0.001; overall *P* <0.0001 one way ANOVA). ANOVA, analysis of variance; BCRP, breast cancer resistant protein; HIF-1α, hypoxia inducible factor 1 α subunit; MTT, 3-[4,5-dimethylthiazol-2-yl]-2,5 diphenyl tetrazolium bromide; n, number; SD, standard deviation.

To determine the functional significance of HIF-1 in LTLTCa cells, the effect of specific inhibition of HIF-1α expression by siRNA on mammosphere formation and cell viability was analyzed (Figure 9). Consistent with our previous study [52], LTLTCa cells formed mammospheres (306 mammospheres/20,000 cells ± 5) (Figure 9B), and this was decreased by BCRP siRNA treatment (64 mammospheres/20,000 cells ± 9; *P* <0.001, one-way ANOVA). HIF-1α siRNA treatment similarly decreased mammosphere formation in LTLTCa cells (101 mammospheres/20,000 cells ± 18), while CoCl_2_ increased formation (500 mammospheres/20,000 cells ± 20) compared to negative control-treated siRNA (*P* <0.001, one-way ANOVA). These results correlated with the effect of HIF-1α inhibition on other genes. HIF-1α siRNA treatment decreased BCRP mRNA (*P* = 0.0377, one-way ANOVA), as well as expression of GAPDH [61] (*P* = 0.0058, one-way ANOVA), another known HIF-1 target gene, and BMI-1 (*P* = 0.0214, one-way ANOVA), another stem cell marker [62] (Figure 9A). HIF-1α siRNA treatment also significantly decreased LTLTCa cell viability (*P* <0.0001, one-way ANOVA) in the presence of increasing concentrations of letrozole (Figure 9C).

#### Effect of HIF-1α upregulation on MCF-7Ca cells

**Figure 10:**
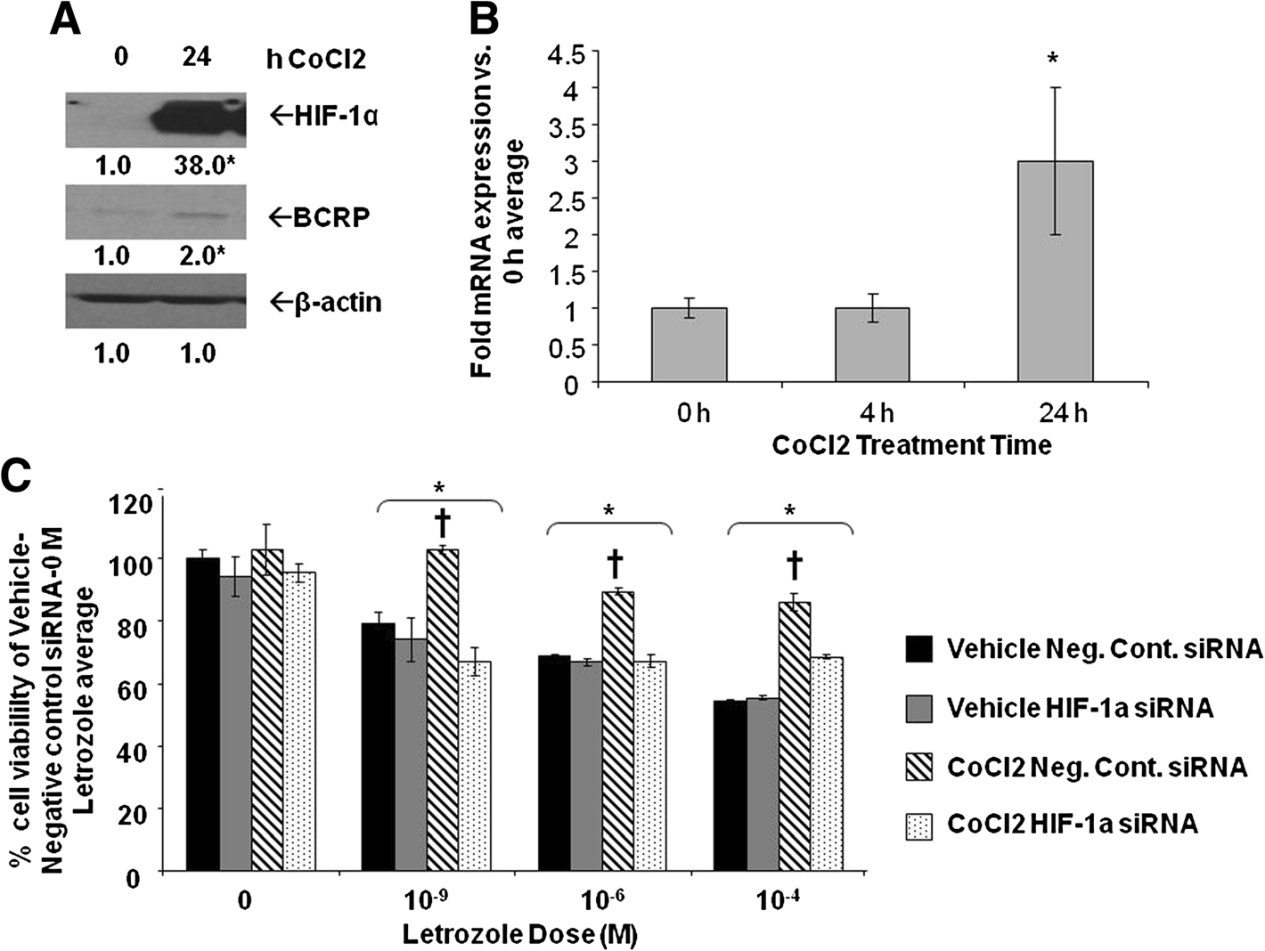
**Effect of CoCl**_2_**on MCF-7Ca protein expression and cell viability. A**-**B**, MCF-7Ca cells were incubated in steroid-free media and then treated with 100 μM CoCl_2_ for 0 to 24 hours. **A)** Total protein was extracted and HIF-1α, BCRP, and β-actin were analyzed by Western blot analysis. Shown are representative blots and overall densitometry results of n = 6 independent cell samples/group. Densitometry results are expressed as mean fold-change in protein levels compared to 0 hours after normalization to β-actin (mean ± SD of n = 4 independent cell samples/group, *versus 0 hours; *P* = 0.0005 for HIF-1a; *P* = 0.0065 for BCRP, two-sided t-test). **B)** Total RNA was extracted and BCRP mRNA and 18S rRNA were analyzed by real-time RT-PCR analysis. Results are expressed as the mean fold-change in mRNA levels compared with 0 hours after normalization to 18S rRNA (mean ± SD, n = 4 independent cell samples/group; *versus 0 hours, *P* <0.001; overall *P* = 0.0002, one-way ANOVA). **C)** Viability of cells was measured by MTT assay after five days of treatment with increasing doses of letrozole following 48 hours pre-treatment with or without 100 μM CoCl_2_ and HIF-1α siRNA. Results are expressed as mean percent of 0 μM letrozole-without CoCl_2_-with negative control siRNA (mean ± SD of n = 6 independent samples/group; *versus vehicle-negative control siRNA-0 μM letrozole, *P* <0.001; † versus vehicle-HIF-1α siRNA and CoCl_2_-HIF-1α siRNA; effect of letrozole *P* <0.0001, effect of pre-treatment (vehicle/CoCl_2_ and negative control siRNA/HIF-1α siRNA) *P* <0.0001, interaction between letrozole dose and pre-treatment *P* <0.0001; two-way ANOVA). ANOVA, analysis of variance; BCRP, breast cancer resistant protein; HIF-1α, hypoxia inducible factor 1 α subunit; MTT, 3-[4,5-dimethylthiazol-2-yl]-2,5 diphenyl tetrazolium bromide; n, number; SD, standard deviation.

To further confirm the physiological role of HIF-1 and BCRP, converse experiments were done to investigate whether MCF-7Ca cells could become more letrozole-resistant with increased HIF-1α. CoCl_2_ was used to increase HIF-1α expression in MCF-7Ca cells and the efficacy of CoCl_2_ was confirmed by Western blot analysis and RT-PCR. Within 24 hours of CoCl_2_ treatment, HIF-1α protein and BCRP mRNA and protein expression were increased in MCF-7Ca cells (Figure 10A-B). Cell viability experiments in the presence of increasing concentrations of letrozole were then performed (*P* <0.0001, one-way ANOVA). Consistent with previous findings from our laboratory [12], MCF-7Ca cells not treated with CoCl_2_ were sensitive to the growth inhibitory effects of letrozole (Figure 10C). Additional treatment of MCF-7Ca cells with CoCl_2_ significantly increased their resistance to letrozole. The effects of CoCl_2_ were attributable to HIF-1, as co-treatment of MCF-7Ca cells with CoCl_2_ and HIF-1α siRNA returned their sensitivity to letrozole. Overall, the physiological experiments on LTLTCa and MCF-7Ca cells indicate that HIF-1 is likely involved in both cancer stem cell characteristics and cell viability.

## Discussion

Prior to this study, AI resistance was associated with increased dependence on growth factors and decreased dependence on ERα. However, the role that such molecular changes play in AI resistance and the mechanism by which they elicit their effects were not known. Novel results from this study demonstrated that nonhypoxic expression of HIF-1 mediates HER2’s effects on letrozole-resistance. Specifically, the HER2-activated PI3K/Akt pathway increases HIF-1α protein synthesis in LTLTCa cells. HIF-1α, in turn, upregulates expression of BCRP and other genes and contributes to letrozole resistance and stem cell characteristics of LTLTCa cells.

Nonhypoxic regulation of HIF-1 expression and activity in LTLTCa cells is due to the HER2-activated PI3K/Akt/mTOR pathway. This is consistent with findings by others indicating hypoxia independent upregulation of HIF-1α in cancer cells by loss of function of tumor suppressor genes and gain of function of oncogenes [27]. The oncogene *HER2/neu*, in particular, has been previously associated with nonhypoxic HIF-1 [24,25]. Laughner *et al*. and Li *et al*. have demonstrated that transfection of HER2 into NIH/3 T3 cells or activation of HER2 in MCF-7 cells led to activation of the PI3K/Akt pathway, and the subsequent increased HIF-1 expression via protein synthesis and HIF-1 transcriptional activity. Previous studies have also demonstrated the importance of mTOR [48,49]. In addition, mTOR has been explored in two randomized trials (BOLERO-2 and TAMRAD) as a potential therapeutic target for overcoming endocrine therapy resistance [63]. Our current study provides evidence that this HER2-PI3K/Akt-mTOR-HIF-1 signaling mechanism can indeed occur endogenously in HER2+ cells (Figures 3, 4, and 8), leads to upregulation of BCRP (Figure 2), and has physiological relevance as well as potential clinical implications (for example, AI resistance; Figures 9 and 10).

Despite providing evidence that HER2 activation of the PI3K/Akt-mTOR pathway regulates HIF-1α, this study cannot completely exclude the involvement of ERα or the MAPK pathway. In addition to overexpressing HER2, LTLTCa cells also have decreased expression of ERα (Figure 3). It is possible that ERα can also regulate non-hypoxic HIF-1α expression in LTLTCa cells. Indeed, ERα- and HIF-1-mediated signaling pathways are known to interact antagonistically [18,19] and cooperatively [20-23]. Although this current study did not directly investigate ERα’s role, the overexpression of HIF-1α observed in both ERα + (Hc7 and Ac1-ExR) and ERα- (LTLTCa) HER2+ breast cancer cell lines, suggests that ERα status may not affect HER2 regulation of non-hypoxic HIF-1α levels. With regard to the MAPK pathway, inhibition of this pathway did not affect HIF-1α expression in LTLTCa cells, but it did decrease BCRP mRNA expression under basal, non-hypoxic conditions. It is possible that the MAPK pathway is involved in phosphorylation of HIF-1α rather than its synthesis. Previous studies have shown that MAPK pathway-mediated phosphorylation of HIF-1α occurs under non-hypoxic conditions and can increase HIF-1α expression and transcriptional activity [64,65]. These results could also indicate the MAPK and PI3K/Akt pathways have very distinct functions in AI-resistant breast cancer cells, regulating different subsets of genes. For example, the MAPK pathway may be involved in HER2 regulation of genes that require activation by phosphorylated ERα^Ser118^. In contrast, the PI3K/Akt pathway may be involved in HER2 regulation of genes that require HIF-1.

Inherent upregulation of HIF-1α protein expression under nonhypoxic conditions is another novel finding in AI-resistant breast cancer. There is precedence for associating HIF-1 expression with drug resistance in different cancer cell types, including chronic myeloid leukemia cells [66], gastric cancer cells [67], non-small cell lung cancer cells [68], and even breast cancer cells [58]. However, these previous cases involved hypoxia-induced, HIF-1α rather than the non-hypoxic HIF-1. Our findings are also consistent with previous clinical evidence that HIF-1α is associated with letrozole resistance. Generali *et al*. demonstrated that increased p-MAPK and HIF-1α protein expression were significant determinants of primary letrozole resistance in breast cancer patients. In contrast, increased ERα and decreased p-MAPK were significant determinants of response to letrozole treatment [56]. The protein expression patterns observed by Generali *et al*. are similar to what is observed in letrozole-resistant LTLTCa and –sensitive MCF-7Ca cells, respectively (Figures 3 and 6). Although these clinical findings involve *de novo* letrozole resistance, they still correlate with, and likely pertain to, our laboratory’s results on acquired letrozole resistance. These results combined suggest that HIF-1 is involved in both *de novo* and acquired AI resistance and, therefore, could be therapeutically targeted to prevent and treat resistance to letrozole and the other AIs.

Lastly, this study indicates that HIF-1 may contribute to letrozole resistance by mediating the effects of HER2 on target genes, such as BCRP. Previous findings by our laboratory had implicated HER2 and BCRP in resistance to the growth inhibitory effects of letrozole and to maintenance of stem cell characteristics in letrozole-resistant breast cancer, and had demonstrated that its expression was dependent on HER2 [35,52], but it was unclear until now how HER2 regulated BCRP. Moreover, HIF-1 may mediate the effects of HER2 on many other genes. Besides, BCRP, other known HIF-1 target genes that may serve as markers of letrozole resistance include: 1) cancer stem cell maintenance markers (*Oct-4, kit ligand, JARID1B*); 2) epithelial-mesenchymal-transition (EMT) markers (*Snail, vimentin*); and 3) invasion markers (*c-Met, endothelin 1, fibronectin, MMP-2 and -4*) [27,69]. Interestingly, another known HIF-1 target gene, *VEGF,* was not upregulated in LTLTCa cells compared to MCF-7Ca cells. It is possible that nonhypoxic HIF-1 expression has different levels of influence on different HIF-1 target genes, particularly those that can be regulated by multiple transcription factors. Indeed BCRP and VEGF both are known to be regulated by additional transcription factors, such as ERα [22,70].

## Conclusions

**Figure 11:**
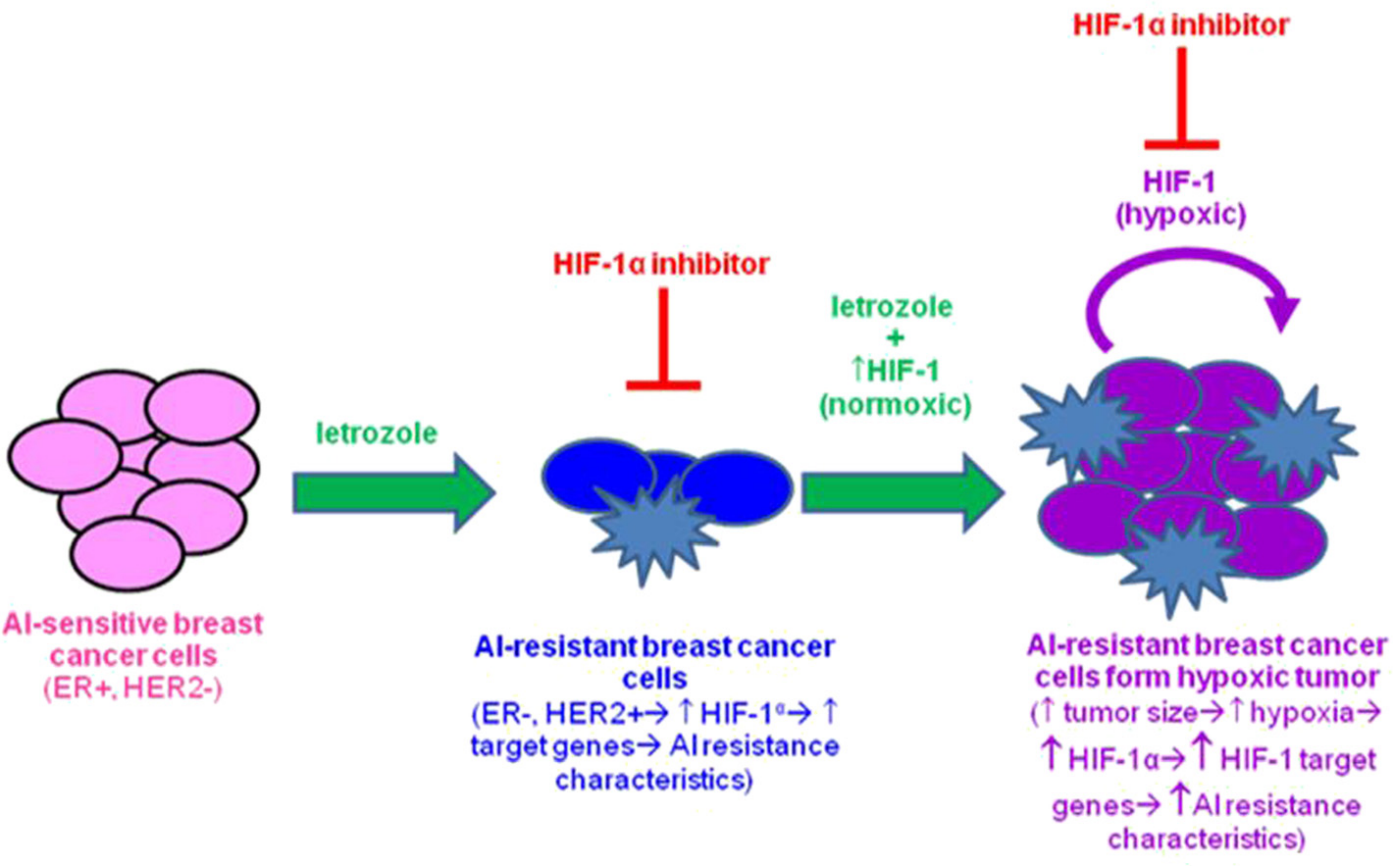
Proposed model of regulation and role of HIF-1α in AI resistant breast cancer.

Overall, this study provides novel evidence that non-hypoxic HIF-1α is inherently expressed in AI-resistant cells, upregulated by HER2-PI3K/Akt-mTOR pathway and is an important factor in letrozole-resistant breast cancer cells, regulating target genes such as *BCRP* and regulating AI responsiveness and cancer stem cell characteristic expression. Thus, HIF-1α could be used as a diagnostic marker and/or therapeutic target. Based on this, a proposed model of acquired AI-resistance may involve the following scenario: under non-hypoxic conditions, when the breast cancer cell population and tumor size have been reduced by letrozole treatment and prior to significant tumor hypoxia, a switch from ERα- to growth factor (for example, HER2)-mediated signaling occurs via PI3K/Akt and mTOR, which leads to increased HIF-1α expression and activation of HIF-1 target genes (for example, *BCRP*) that contribute to AI resistance (Figure 11). Consequently, inhibition of HIF-1 expression and/or activity would prolong cancer cell sensitivity to AIs and prevent recurrence and metastasis. Indeed, a number of anti-cancer drugs in clinical use are also known to inhibit HIF-1 [27]. They include HER2 inhibitor, trastuzumab [24] and lapatinib. Furthermore, as demonstrated in this study that HIF-1 is regulated mainly via the PI3K/Akt/mTOR pathway, inhibition of the downstream affecter of this pathway using mTOR inhibitors, such as rapamycin, temsirolimus/CCI-779 and everolimus/RAD-001, can also be considered [24,71-73]. There is also EZN-2968, a specific HIF-1α mRNA inhibitor, shown to reduce cancer cell viability and xenograft tumor growth, which is currently under phase I clinical trial [74]. Any of these anti-cancer drugs could now potentially, based on the evidence provided by this study, be applied to the prevention and treatment of AI-resistant breast cancer.

## Abbreviations

AC1: MCF-7 cells transfected with the aromatase gene by the laboratory of Dr. Brodie; AC1-ExR: long-term exemestane treated AC1 cells; AI: aromatase inhibitor; Akt: also known as protein kinase B; ANOVA: analysis of variance; BCRP: breast cancer resistance protein; CDT: charcoal-dextran treated; ChIP: chromatin immunoprecipitation; (D)MEM: (Dulbecco’s) modified Eagle’s medium; EGFR: epidermal growth factor receptor; ERα: estrogen receptor alpha; FBS: fetal bovine serum; Hc7: MCF-7 cells transfected with HER2 gene by the laboratory of Dr. Brodie; HER2: human epidermal growth factor receptor 2; HIF-1: hypoxia inducible factor 1; HIF-1α: hypoxia inducible factor 1 α subunit; HRE: hypoxia-response element; LTLTCa: long-term letrozole treated MCF-7Ca cells; MAPK: mitogen-activated protein kinase; MCF-7Ca: MCF-7 cells transfected with the aromatase gene by the laboratory of Dr. Chen; mTOR: mammalian target of rapamycin; PBS: phosphate-buffered saline; PI3K: phosphatidylinositide 3-kinase; PRF: phenol red-free; ORF: open reading frame; siRNA: small interfering RNA; RT-PCR: reverse transcriptase-polymerase chain reaction; VEGF: vascular endothelial growth factor.

## Competing interests

The authors declare that they have no competing interests.

## Authors’ contributions

AK conceived of and designed the study, performed all experiments, and drafted the manuscript. RG participated in RT-PCR in Figure 1A and western blot 1B. AS participated in densitometry analysis of Figure 3A and helped to draft the manuscript. SC coordinated obtaining lapatinib and trastuzumab and participated in discussions of study design. GS participated in the analysis and interpretation of Western blot data for Figures 3, 4, and 8 and statistical analyses for Figures 1B and 2C. PS participated in the mammosphere assay and selection of cancer stem cell markers in Figure 9A. OG performed statistical analyses for Figures 1B and 2C. SK performed statistical analyses for Figures 1B and 2C. AB participated in the design of the study, interpretation of all results, and the critical review and revision of the manuscript. All authors read and approved final manuscript.

## Supplementary Material

Additional file 1: Table S1.1 Test results conducted to compare mean differences of normalized, log-transformed HIF-1α mRNA expression at various time points of actinomycin D for LTLTCa and MCF-7Ca cells. Shown are estimated mean differences in log transformed, normalized HIF-1α mRNA expression between different timepoints of actinomycin D treatment. Table results are estimated mean differences in log transformed, normalized HIF-1α mRNA expression between different timepoints of actinomycin D treatment calculated from the same data as shown Figure 1B data, which indicates trend over time. Pre-specified timepoints compared within each cell line were 0 versus 2 hours, 2 versus 4 hours, or 4 versus 16 hours. Data were analyzed by linear mixed effect model adjusting for experiment, cell line, and cell line*time interaction mRNA. Fixed effects for time, experiment, cell lines and interactions between time and cell lines were determined (means ± SD of n = 6 independent samples/group; *P* <0.001 for effect of cell line, time, their interaction and experiment). NS, not significant, *P* >0.05. **Table S1.2.** Test results conducted to compare mean differences of normalized, log-transformed BCRP mRNA expression at various time points of actinomycin D for both LTLTCa and MCF-7Ca cells. Table results are estimated mean differences in log transformed, normalized BCRP mRNA expression between different timepoints of actinomycin D treatment calculated from the same data as shown in Figure 2C. Pre-specified timepoints compared within each cell line were 0 versus 2 hours, 2 versus 4 hours, or 4 versus 16 hours. Data were analyzed by linear mixed effect model adjusting for experiment, cell line, and cell line*time interaction mRNA. Fixed effects for time, experiment, cell lines and interactions between time and cell lines were determined (means ± SD of n = 6 independent samples/group; *P* <0.001 for effect of time and cell line*time interaction). NS, not significant, *P* >0.05. Click here for file
