# Supplementary figures and images for: Nonhypoxic regulation and role of hypoxia-inducible factor 1 in aromatase inhibitor resistant breast cancer
Source: Breast Cancer Res. 2014 Jan 29;16(1):R15. doi: 10.1186/bcr3609 (PMC3978891; doi:10.1186/bcr3609)

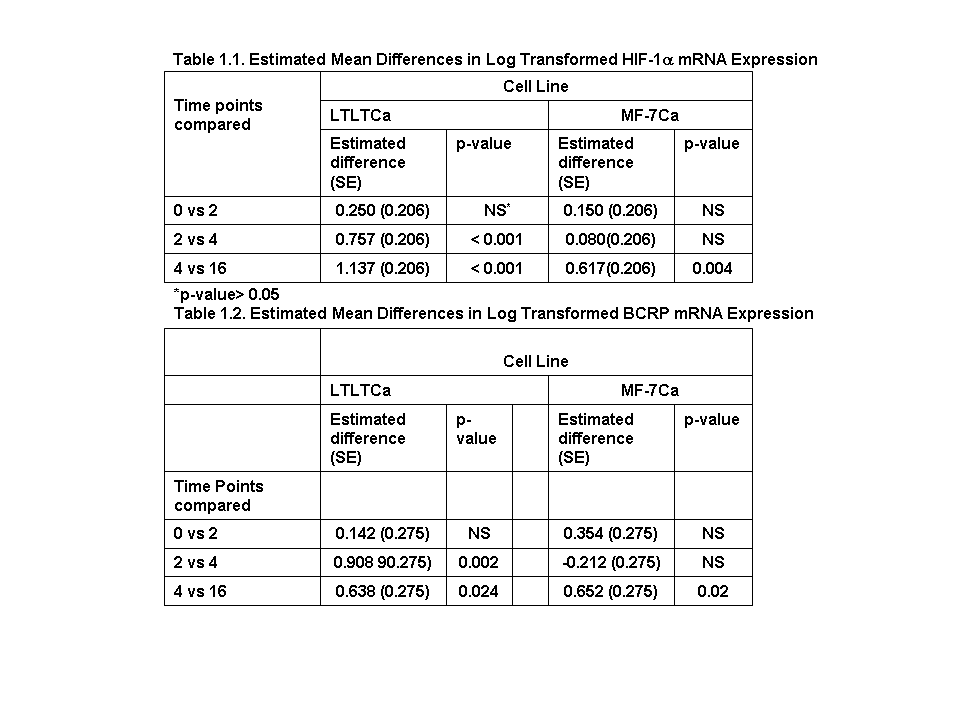

Supplement: Additional file 1: Table S1.1 — Test results conducted to compare mean differences of normalized, log-transformed HIF-1α mRNA expression at various time points of actinomycin D for LTLTCa and MCF-7Ca cells. Shown are estimated mean differences in log transformed, normalized HIF-1α mRNA expression between different timepoints of actinomycin D treatment. Table results are estimated mean differences in log transformed, normalized HIF-1α mRNA expression between different timepoints of actinomycin D treatment calculated from the same data as shown Figure 1B data, which indicates trend over time. Pre-specified timepoints compared within each cell line were 0 versus 2 hours, 2 versus 4 hours, or 4 versus 16 hours. Data were analyzed by linear mixed effect model adjusting for experiment, cell line, and cell line*time interaction mRNA. Fixed effects for time, experiment, cell lines and interactions between time and cell lines were determined (means ± SD of n = 6 independent samples/group; P <0.001 for effect of cell line, time, their interaction and experiment). NS, not significant, P >0.05. Table S1.2. Test results conducted to compare mean differences of normalized, log-transformed BCRP mRNA expression at various time points of actinomycin D for both LTLTCa and MCF-7Ca cells. Table results are estimated mean differences in log transformed, normalized BCRP mRNA expression between different timepoints of actinomycin D treatment calculated from the same data as shown in Figure 2C. Pre-specified timepoints compared within each cell line were 0 versus 2 hours, 2 versus 4 hours, or 4 versus 16 hours. Data were analyzed by linear mixed effect model adjusting for experiment, cell line, and cell line*time interaction mRNA. Fixed effects for time, experiment, cell lines and interactions between time and cell lines were determined (means ± SD of n = 6 independent samples/group; P <0.001 for effect of time and cell line*time interaction). NS, not significant, P >0.05. [file bcr3609-S1.tiff]
